# Multiplex detection of nine food-borne pathogens by mPCR and capillary electrophoresis after using a universal pre-enrichment medium

**DOI:** 10.3389/fmicb.2015.01194

**Published:** 2015-11-03

**Authors:** Germán Villamizar-Rodríguez, Javier Fernández, Laura Marín, Juan Muñiz, Isabel González, Felipe Lombó

**Affiliations:** ^1^Research Unit “Biotechnology and Experimental Therapy Based in Nutraceuticals-BITTEN,” Instituto Universitario de Oncología del Principado de Asturias, Universidad de OviedoOviedo, Spain; ^2^Área de Microbiología, ALCE Calidad S.L. LlaneraLlanera, Spain; ^3^Departamento I+D+i, Industrias Lácteas Asturianas, S.A. (Reny Picot)Navia, Spain

**Keywords:** food pathogens, mPCR, capillary electrophoresis, universal pre-enrichment

## Abstract

Routine microbiological quality analyses in food samples require, in some cases, an initial incubation in pre-enrichment medium. This is necessary in order to ensure that small amounts of pathogenic strains are going to be detected. In this work, a universal pre-enrichment medium has been developed for the simultaneous growth of *Bacillus cereus, Campylobacter jejuni, Clostridium perfringens, Cronobacter sakazakii, Escherichia coli, Enterobacteriaceae* family (38 species, 27 genera), *Listeria monocytogenes, Staphylococcus aureus, Salmonella* spp. (two species, 13 strains). Growth confirmation for all these species was achieved in all cases, with excellent enrichments. This was confirmed by plating on the corresponding selective agar media for each bacterium. This GVUM universal pre-enrichment medium could be useful in food microbiological analyses, where different pathogenic bacteria must be detected after a pre-enrichment step. Following, a mPCR reaction for detection of all these pathogens was developed, after designing a set of nine oligonucleotide pairs from specific genetic targets on gDNA from each of these bacteria, covering all available strains already sequenced in GenBank for each pathogen type. The detection limits have been 1 Genome Equivalent (GE), with the exception of the Fam. *Enterobacteriaceae* (5 GEs). We obtained amplification for all targets (from 70 to 251 bp, depending on the bacteria type), showing the capability of this method to detect the most important industrial and sanitary food-borne pathogens from a universal pre-enrichment medium. This method includes an initial pre-enrichment step (18 h), followed by a mPCR (2 h) and a capillary electrophoresis (30 min); avoiding the tedious and long lasting growing on solid media required in traditional analysis (1–4 days, depending on the specific pathogen and verification procedure). An external testing of this method was conducted in order to compare classical and mPCR methods. This evaluation was carried out on five types of food matrices (meat, dairy products, prepared foods, canned fish, and pastry products), which were artificially contaminated with each one of the microorganisms, demonstrating the equivalence between both methods (coincidence percentages between both methods ranged from 78 to 92%).

## Introduction

*Bacillus cereus, Campylobacter jejuni, Clostridium perfringens, Cronobacter sakazakii, Escherichia coli*, bacteria from the *Enterobacteriaceae* family (38 species, 27 genera), *Listeria monocytogenes, Staphylococcus aureus*, and *Salmonella* spp. are the most common bacterial pathogens whose detection is mandatory in many countries, depending on the specific food matrix (Chen and Jiang, 2014). Most national food quality legislations define, for a given food matrix, the maximum level of allowed bacterial species and CFUs per gram. In most cases, these classical microbiology detection methods require a pre-enrichment stage using a non-selective medium (Crowley et al., 2011). This is true in the case of certain pathogens, where the number of pathogen cells (potentially present in that food and causing clinical symptoms) can be very low (*C. jejuni*), or when those pathogens can cause potentially fatal systemic syndromes (*Cr. sakazakii* in newborns, *E. coli* O157:H7, *L. monocytogenes* and *Salmonella* spp.) [Lecuit, 2005; Lefebvre et al., 2009; Kalischuk and Buret, 2010; Iebba et al., 2012; Swart and Hensel, 2012; Centers for Disease Control and Prevention (CDC), 2013; Hariri et al., 2013; Grisaru, 2014; Holý and Forsythe, 2014].

In all these cases, the legally established detection methods need a pre-enrichment step for these potential pathogens (from the food matrix) in order to facilitate subsequent detection steps (Dusch and Altwegg, 1993). The objective of this pre-enrichment step is the detection of putative present pathogenic cells, which in some cases, can have a minimum sensitivity threshold of 1–10 CFUs (ISO 22118:2011). So, during several hours (usually 18–24 h), these bacteria will multiply in the pre-enrichment medium, giving rise to thousands or millions of descendants. This will therefore facilitate their final positive detection (Chap et al., 2009; Kotzekidou, 2013).

In our case, it was impossible to pre-enrich at the same time all nine types of pathogens by using any of the available culture media for these purposes. Most of these nine pathogens can grow in usual pre-enrichment media as for example BPW, but others like *C. jejuni* or *St. aureus* do not. For example, *C. jejuni* usually is grown in Bolton Growth, Oxoid CM0983 (Murinda et al., 2004; Jokinen et al., 2012); whereas *St. aureus* is usually grown in Baird-Parker, Oxoid CM0961 (Saito et al., 2011). With the aim to solve this issue, a universal pre-enrichment medium was designed, in which it was demonstrated a good growth rate for each one of the nine types of bacteria.

Also, reference detection protocols usually require a second phase, called enrichment, in which a selective medium is used. This phase allows only the growth of a specific pathogen, after the initial multi-species pre-enrichment. These two phases may themselves constitute up to 48–72 h, depending on the pathogen to be detected (Iebba et al., 2012). It is, therefore, of enormous social, economic, and health importance to have rapid methods for determining the presence/absence of these food-borne pathogens in food matrices of any type, as this can stop the health problem before it extends and gets converted into clinical cases. This would allow great savings from the industrial point of view and from the perspective of the health authorities.

For example, for *Salmonella*, this pre-enrichment step, under current legal methods for detection of food-borne pathogens [as Commission Regulation (EU) 365/2010 and 2073/2005; FDA Food Code 2009 in the USA; Australia and New Zealand's Food Standards Code 1.6.1; etc.] follows the international ISO 6579:2005 method for its detection in food, in which 25 g food samples are first incubated in Buffered Peptone Water (BPW), for 18 h at 37°C. Afterwards, an aliquot of the sample is then incubated in parallel in selective Rappaport–Vassiliadis broth (with soy) during 24 h at 41.5°C and in Muller–Kauffmann broth (with tetrathionate and novobiocin) 24 h at 37°C [International Standards Organization (ISO), 2005]. These cultures are finally plated on selective Xylose lysine deoxycholate agar plates and incubated 24 h at 37°C (Jasson et al., 2011; Sánchez et al., 2012). This method requires excessive manipulation of samples or cultures; which can become a tedious and expensive procedure in laboratory materials, pre-cultures preparation, culture media inoculation (Petri dishes, test tubes, etc.), analysis of results, and new rounds of inoculation of doubtful colonies to confirm the suspected pathogenic isolated species (Jokinen et al., 2012). BPW is not suitable for all food-borne pathogens, and therefore, other pre-enrichment media have been developed, as Bolton Broth (BB) and Reinforced *Clostridium* Medium (RCM) (Tansuphasiri et al., 2005; Jokinen et al., 2012).

Nowadays, research on molecular biology techniques provides experimental approaches, like ELISA immunoassay (Shim et al., 2008), and tests as VITEK and VIDAS, which allows the detection of a single pathogen type in one experiment. But these approaches still require the previous isolation of pure colonies of the species to be tested (Valero et al., 2007; Temelli et al., 2012).

Conventional PCR allows detection of a single specific pathogen species or strain from a food matrix sample, based on a specific DNA sequence, avoiding an initial colony isolation step. Many PCR detection protocols have been developed in the last years for all the above-mentioned food-borne pathogens, as for *Salmonella* in dairy products (Stevens and Jaykus, 2004) or *Cr. sakazakii* in infant milk formula (Cai et al., 2013). Multiplex PCR (mPCR) allows amplification of several target DNA sequences from several food-borne pathogens, which can be visualized for example by agarose gel electrophoresis. mPCR protocols have been described for three species of *Campylobacter* genus (Asakura et al., 2008), or for simultaneous detection of EHEC, *Salmonella* spp., *St. aureus* and *L. monocytogenes* from plant derived fermented food matrices (Park et al., 2006). Visualization of the resulting PCR products can be achieved also by capillary gel electrophoresis, saving analysis time, as it has been described for *St. aureus, L. monocytogenes*, and *Salmonella* spp. from meat products (Cremonesi et al., 2014). Another possibility is to use qRT-PCR for detection of these pathogens. For example, TaqMan and molecular beacon based assays have been used on multiplex detection of 23, 8, and 3 food-borne pathogens in one single reaction (Suo et al., 2010; Cremonesi et al., 2014; Hu et al., 2014).

In this work, we describe the formulation of a universal pre-enrichment medium, GVUM (“Germán Villamizar Universal Medium”), which allows growth of nine types of food-borne bacterial pathogens (seven species, one genus, and one family), whose detection is mandatory under most national laws. This work also describes that it is possible to grow all pathogens, individually or simultaneously, in GVUM, and they can be isolated from 19 types of food matrices by using GVUM, including diverse types as dairy, meat, vegetables, fish, or cooked food. Then, we describe the design of a mPCR detection system for these nine pathogens, all at the same time. This method comprises a non-selective pre-enrichment step (18 h), followed by a mPCR (2 h) and a capillary electrophoresis (30 min). This is in contrast with traditional methods, where detection of present species is done by plating pre-enrichment on solid media, an extra step requiring between 24 and 96 h, depending on the specific pathogen. All these pathogens are of great economic and epidemiological importance in most countries, and this detection system covers food poisoning and food infections from all main types of food matrices (five different families were spiked during external evaluation).

## Materials and methods

### Bacterial strains, growth conditions, and maintenance

Sixty seven strains of one family, one genus, and seven species of food-borne pathogens were acquired at American Type Culture Collection (ATCC), Colección Española de Cultivos Tipo (CECT), and Deustche Sammlung von Mikroorganismen und Zellkulturen (DSMZ), to create a collection. This was used to execute the necessary experiments in order to develop and to optimize the GVUM universal medium. The bacterial strains and the corresponding culture conditions (temperature; culture media; aeration) are described in Table 1. A stock was created preserving aliquots of each culture at −80°C with 25% of glycerol in the corresponding liquid culture medium to avoid freezing damage. GVUM composition is: BPW (20 g/L), mannitol (20 g/L), hemolyzed and defibrinated horse blood (50 mL/L), commercial *Campylobacter* Growth Supplement (4 mL/L).

**Table 1:** **List of target species in this work, including ***Enterobacteriaceae*** and negative control species**.

**Strain**	**Code**	**Culture medium**	**Temperature °C**	**[O_2_]**
*Bacillus cereus*	CECT 131	BBCA	30°	A
*Campylobacter jejuni*	ATCC 49943	KCA	42°	MA
*Clostridium perfringens*	CECT 376T	RCM	37°	AN
*Cronobacter sakazakii*	ATCC BAA-894	BESA	37°	FAN
*Escherichia coli* DH10B	Invitrogen	EMBA	37°	FAN
*Listeria monocytogenes*	CECT 911	BHI, BLMA	37°	A
*Staphylococcus aureus*	CECT 240	MSA	37°	FAN
*Salmonella bongori*	ATCC 43975	KIA	37°	FAN
*Salmonella enterica* var. *arizonae*	CECT 4395	KIA	37°	FAN
*Salmonella enterica* var. *houtenae*	CECT 5326	KIA	37°	FAN
*Salmonella enterica* var. *salamanae*	CECT 4000T	KIA	37°	FAN
*Salmonella enterica enterica* var. *tiphy*	CECT 725	KIA	37°	FAN
*Salmonella enterica enterica* var. *Tiphymurium*	CECT 443	KIA	37°	FAN
*Salmonella enterica enterica* var. *paratyphi*	CECT 698	KIA	37°	FAN
*Salmonella enterica enterica* var. *paratyphi*	CECT 554	KIA	37°	FAN
*Salmonella enterica enterica* var. *enteritidis*	CECT 556	KIA	37°	FAN
*Salmonella enterica enterica* var. *infantis*	CECT 707	KIA	37°	FAN
*Salmonella enterica enterica* var. *choleraesuis*	CECT 724	KIA	37°	FAN
*Salmonella enterica enterica* var. *dublin*	CECT 4152	KIA	37°	FAN
*Salmonella enterica enterica* var. *virchow*	CECT 4154	KIA	37°	FAN
**FAMILY** ***ENTEROBACTERIACEAE***				
*Budvicia aquatica*	DSM 5075	TSA-VRBGA	26°	FAN
*Buttiauxella agrestis*	DSM 4586	NAI-VRBGA	30°	FAN
*Cedecea davisae*	CECT 842T	NAI-VRBGA	28°	FAN
*Citrobacter youngae*	CECT 5335T	LA-VRBGA	37°	FAN
*Edwardsiella tarda*	CECT 849T	TSA-VRBGA	26°	FAN
*Erwinia chrysanthemi*	CECT 509	NAI-VRBGA	37°	FAN
*Ewingella americana*	CECT 859T	TSA-VRBGA	26°	FAN
*Klebsiella pneumoniae*	CECT 144	TSA-VRBGA	37°	FAN
*Kluyvera cryocrescens*	CECT 862T	NAI-VRBGA	37°	FAN
*Leclerciaader carboxylata*	DSM 5077	NAI-VRBGA	37°	FAN
*Leminorella grimontii*	DSM 5078	NAI-VRBGA	37°	FAN
*Morganella morganii*	CECT 173T	NAI-VRBGA	37°	FAN
*Pantoea ananatis*	CECT 4858T	TSA-VRBGA	26°	FAN
*Pectobacterium carotovorum*	CECT 225T	TSA-VRBGA	26°	FAN
*Plesiomonas shigelloides*	CECT 597	NAI-VRBGA	37°	FAN
*Proteus vulgaris*	CECT 165	NAI-VRBGA	37°	FAN
*Providencia stuartii*	CECT 866T	NAI-VRBGA	37°	FAN
*Rahnella aquatilis*	DSM 4594	NAI-VRBGA	30°	FAN
*Raoultella planticola*	CECT 843T	NAI-VRBGA	30°	FAN
*Serratia marcescens*	CECT 854	TSA-VRBGA	26°	FAN
*Shigella dysenteriae*	CECT 584	TSA-VRBGA	37°	FAN
*Tatumella ptyseos*	CECT 869T	TSA-VRBGA	30°	FAN
*Hafnia alvei*	CECT 157	TSA-VRBGA	30°	FAN
*Yersinia enterocolitica*	CECT 4054	TSA-VRBGA	26°	FAN
**NEGATIVE CONTROLS**				
*Bacillus badius*	CECT 17T	NAI	30°	A
*Bacillus pumilus*	CECT 29T	NAI	30°	A
*Bacillus subtilis*	CECT 35	NAI	30°	A
*Campylobacter coli*	ATCC 49941	CBA	37°	MA
*Campylobacter mucosalis*	DSM 21682	CBA	37°	AN
*Campylobacter helveticum*	ATCC 51209	CBA	35°	MA
*Clostridium beijerinckii*	CECT 508	LVB	37°	AN
*Clostridium butyricum*	CECT 361T	LVB	37°	AN
*Enterobacter aerogenes*	CECT 684T	NAI	30°	FAN
*Enterobacter cloacae*	CECT 194T	NAI	30°	FAN
*Enterobacter gergoviae*	CECT 857T	NAI	37°	FAN
*Escherichia fergusonii*	DSM 13698	NAI	37°	FAN
*Escherichia hermannii*	DAM 4560	NAI	37°	FAN
*Escherichia intermedia*	ATCC 21073	NAI	37°	FAN
*Listeria grayi*	CECT 931T	BHI	37°	A
*Listeria innocua*	CECT 910T	BHI	37°	A
*Listeria ivanovii*	CECT 5379	BHI	37°	A
*Staphylococcus epidermidis*	CECT 231	NAI	37°	FAN
*Staphylococcus haemolyticus*	CECT 4900T	NAI	37°	FAN
*Staphylococcus simulans*	CECT 4538T	NAI	37°	FAN

*Aeration culture conditions for these strains are also indicated in next paragraph with these abbreviations: A, aerobic; AN, anaerobic; FAN, facultative anaerobic; MA, microaerophilic. Culture media for testing growth and for quantification of these strains were the following ones (see species paragraph below): BBCA, Brilliance Bacillus cereus Agar; BESA, Brilliance Enterobacter sakazakii agar; BHI, Brain-Heart Infusion; BLMA, Brilliance Listeria Agar; EMBA, Eosin Methylene Blue Agar (Levine); KCA, Karmali Campylobacter Agar; KIA, Klieger Iron Agar; LA, Luria-Bertani Agar; LVB, Liver Broth; MSA,Salt mannitol agar; NAI, Nutrient Agar I; RCM, Reinforced Clostridium Medium; VRBGA, Violet Red-Bile-Glucose Agar. For multispecies experiments, Salmonella detection and quantification was carried out using a modification of KIA, containing methylene blue (0.1 g/l), in order to avoid L. monocytogenes overgrowth on the same plates, and therefore to facilitate CFUs counting*.

### Food samples spiking

In order to test growth of the nine different pathogens in the universal pre-enrichment culture medium (GVUM), 19 types of food samples (bovine grounded meat, fresh chicken, fresh fish, canned fish, fish *consommé*, fresh and cured sausages, raw milk, yogurt, two different infant milk formulae (with and without plant starches), cheese, cream, raw and cooked eggs, pastry, honey, dairy dessert, and vegetables) were contaminated with 300 CFUs/g (total 30,000 CFUs/100 g batch) of each bacterial strain, in order to test the growth of each individual microorganism in GVUM medium. In a separate experiment, we decided to test if spiking of the bacteria at different levels has any influence in their growth during enrichment, and if some competition between species occurs. To test this, two other enrichments (duplicate experiments) were carried out: one is using 10 CFUs/g (total 1000 CFUs/100 g batch) of *Salmonella* sp. (as example of *Enterobacteriaceae*) or *L. monocytogenes*, plus 1000 CFUs/g (total 100,000 CFUs/100 g batch) of the other seven microorganisms in each case. The food matrices list was extracted from the NordVal system, and covers almost all possible food matrices types. Control experiments were also carried out by using only 100 g GVUM (without added matrix), and the corresponding 300 CFUs/g of each individual bacterial species, in order to test if any used food matrix could inhibit growth of some of the individual pathogens. No inhibition due to food matrix was detected in any case, as control experiments using only 100 g GVUM medium rendered bacterial counts between 10^8^ and 10^9^ CFUs/mL, and these results are very similar to those experiments including added food matrix (see Table 2).

**Table 2:** **Number of CFUs (average and SD) obtained for each bacterial species after individual or multispecies pre-enrichment on GVUM**.

**Microorganism**	**CFUs after individual enrichment (SD)**	**CFUs after mixed enrichment (SD)**	**CFUs when *Salmonella* sp. at low initial concentration (SD)**	**CFUs when *L. monocytogenes* at low initial concentration (SD)**
*B. cereus*	4.20 × 10^9^ (1.90 × 10^9^)	7.93 × 10^8^ (1.64 × 10^8^)	1.29 × 10^7^ (6.36 × 10^5^)	5.40 × 10^6^ (1.13 × 10^6^)
*C. jejuni*	4.38 × 10^9^ (3.50 × 10^9^)	1.47 × 10^8^ (9.21 × 10^7^)	1.90 × 10^7^ (1.13 × 10^7^)	2.85 × 10^7^ (1.34 × 10^7^)
*Cl. perfringens*	8.20 × 10^9^ (6.77 × 10^9^)	2.04 × 10^9^ (1.26 × 10^9^)	2.48 × 10^7^ (7.78 × 10^5^)	6.05 × 10^7^ (2.90 × 10^7^)
*Cr. sakazakii*	5.47 × 10^9^ (3.23 × 10^9^)	5.03 × 10^8^ (2.45 × 10^8^)	2.75 × 10^8^ (1.20 × 10^7^)	5.18 × 10^8^ (5.97 × 10^8^)
*E. coli*	6.27 × 10^9^ (3.02 × 10^9^)	1.68 × 10^9^ (6.11 × 10^8^)	7.70 × 10^8^ (5.56 × 10^7^)	1.62 × 10^8^ (2.19 × 10^7^)
*L. monocytogenes*	6.90 × 10^8^ (1.40 × 10^8^)	4.43 × 10^7^ (1.43 × 10^7^)	2.14 × 10^7^ (1.70 × 10^6^)	4.70 × 10^5^ (2.12 × 10^5^)
*S. enterica*	6.70 × 10^9^ (1.60 × 10^9^)	2.76 × 10^9^ (1.32 × 10^9^)	2.21 × 10^8^ (6.36 × 10^6^)	1.09 × 10^9^ (2.69 × 10^8^)
*St. aureus*	5.50 × 10^8^ (3.86 × 10^8^)	3.05 × 10^7^ (6.23 × 10^6^)	3.10 × 10^7^ (9.90 × 10^6^)	3.20 × 10^7^ (3.20 × 10^7^)

*Final data are the average of three independent experiments. SD, standard deviation. Two last columns show same multispecies experiments, but using in one case 10 CFUs/g for Salmonella sp. and 1000 CFUs/g for the other microorganisms, or alternatively 10 CFUs/g for L. monocytogenes and 1000 CFUs/g for the other microorganisms, before incubation*.

Ten gram of each food matrix was weighted into a Whirl-Pak Sampling Bag (Nasco) with an MXX-061 scale (Denver Instrument). Then, 90 g of the new formulated universal pre-enrichment medium (GVUM) were added. GVUM medium is composed as follows: BPW (20 g/L, Merck), hemolyzed and defibrinated horse blood (50 mL/L, Oxoid SR0048C), mannitol (20 g/L, Merck), and commercial *Campylobacter* growth supplement (4 mL/L, Oxoid SR0232, obtained from a registered company registered with certificate number FS28907). Then, the samples were ground using a Stomacher 80 (Seward) during 30 s.

Finally, bags were incubated at 37°C for 18 h. After this time, 100 μl of nine decimal dilutions of these cultures (1 mL samples) were plated on the recommended solid medium for each microorganism (see above) and incubated under the optimal conditions (see above) to test pathogen recovery. For growth comparison purposes between GVUM and BPW media, all experiments were carried out three times, and average CFUs values used as results. Also, from each of these cultures (19 types of food matrixes added to GVUM medium, and the control using only GVUM as culture medium), 1 mL of the corresponding culture was used for extracting gDNA using the PrepMan Ultra Reagent (Life Technologies). This gDNA was used for testing PCR amplification of the corresponding target gene for each microorganism, including in all cases positive control wells where the amplification material was pure gDNA from pure cultures of the corresponding species in its recommended culture medium (Table 1), and including also in all cases negative control wells were the amplification material was no DNA.

Also, in order to test possible growth interferences between any of the nine pathogens, these spiking experiments were repeated (GVUM alone, and GVUM with each one of the 19 food matrices), but inoculating at the same time 300 CFUs/g (total 30,000 CFUs/100 g batch) of each pathogen species.

Finally, five types of food matrices (bovine grounded meat, canned fish, canned baked beans, vanilla cake, and powder milk) were contaminated, following the previous protocol, with all nine pathogens (or each one individually), in order to isolate gDNA for PCR reactions from 1 mL of these spiked cultures.

### Genomic DNA extraction

gDNA template for PCR positive controls was extracted from pure bacterial cultures using the DNeasy Blood and Tissue Kit (QIAGEN). The gDNA from spiked food samples was extracted with PrepMan Ultra Reagent (Life Technologies). Both types of gDNA were stored at −20°C.

### Primer design for PCR target genes

Target sequences for each pathogen were chosen based on the specificity of each one within its corresponding species, genus or family. Genes coding for variable features, like some toxins or specific virulence factors, were avoided as possible trying to maintain the universality of the primer sets for each one of the targeted pathogens. All the selected sequences were obtained from GenBank (NCBI) and the analysis for specificity and potential cross detection with other pathogens was performed with Blastn and Blastp tools (Altschul et al., 1997). From the protein sequence of each one of these gene targets, only conserved domains for many different strains of the corresponding pathogen were identified, using the online software Blastp.

Once the target sequences were selected, a pair of specific and compatible oligonucleotides was designed for each microorganism using the online tool Primer3 (Rozen and Skaletsky, 1998). The mPCR amplicon size was selected, considering at least a 10 base pairs size difference between all nine PCR products. The corresponding quality tests for each designed oligonucleotide were performed with the software FastPCR (Kalendar et al., 2011) to avoid primer dimerization and other unwanted properties.

### PCR conditions and optimization

For all the PCR and mPCR reactions, AmpliTaq Gold 360 Master Mix (Life Technologies) was used (final concentration 1 ×). This mix includes the thermostable polymerase, dNTPs, MgCl_2_, and the recommended buffer. The PCR reaction mix included also UP and RP primers (1 μM final concentration) and gDNA. Final volume was 25 μl. Best PCR conditions such as reagents' concentrations and annealing temperature, were determined individually for each oligonucleotide pair, testing 16 combinations of primer concentrations (from 0.4 to 1 μM in any given UP/RP primer combination) and annealing temperatures from 52 to 60°C, in order to choose the better conditions for multiplex optimization. All PCR reactions were performed in a Veriti 96-well Thermal Cycler (Life technologies).

As the starting point of the multiplex optimization process, only one oligonucleotide pair for each microorganism was tested. Then, once the combination with the best performance was determined for this experiment, new oligonucleotide pairs were added subsequently and tested with the best combinations of temperature and concentration obtained in previous steps: denaturation step (95°C, 180 s, 1 cycle); 35 cycles of denaturation step (95°C, 30 s), plus annealing step (56°C, 60 s), plus elongation step (72°C, 90 s), and a final elongation step (72°C, 420 s).

Once the positive amplification peaks for all these targets were obtained, in order to reduce competition between oligonucleotides and improve reproducibility, final concentrations at mPCR reactions were manually adjusted until obtaining the best possible amplification (Table 3).

**Table 3:** **List of oligonucleotides designed for mPCR and final oligonucleotides concentrations in the mPCR**.

**Primer**	**Sequence (5′ – 3′)**	**Target**	**Product size (bp)**	**Concentration**
	**1**. ***B. cereus***			
GVR-BC-Mpx-up	GCGTACTGAGTTAGAGAACGGT	*cerAB*	132	1 μM
GVR-BC-Mpx-rp	TTTGCTTGCTTTGCATACGGA	(phospholipase and sphingomyelinase)		
	**2**. ***C. jejuni***			
GVR-CJ-Mpx-up	GAGTGAGGCGAAATTCCAAC	*pldA*	251	2.5 μM
GVR-CJ-Mpx-rp	TCTCATCTCCCTTGCCATTG	(phospholipase)		
	**3**. ***Cl. Perfringens***			
GVR-CP-Mpx-up	TGGGAAAGTTCTTTCAACACC	*rplS*	116	2 μM
GVR-CP-Mpx-rp	GAGAAAGAATCCAAGTATTCGAAGG	(ribosomal protein)		
	**4**. ***Cr. sakazakii***			
GVR-CS-Mpx-up	TGGCATCATCAACACTTTCGT	*kpsT*	196	0.5 μM
GVR-CS-Mpx-rp	TCGACTACTACCTGGTGGACG	(capsular transport protein)		
	**5**. ***E. coli***			
GVR-EC-Mpx-up	GTTGGTGGGAAAGCGCGTTACA	*uidA*	70	0.4 μM
GVR-EC-Mpx-rp	CGTTAAAACTGCCTGGCACAG	(β-glucuronidase)		
	**6. Fam*****. Enterobacteriaceae***			
GVR-EB-Mpx-up	TCAGAGTTCCCGAAGGCACTC	*16S rRNA*	77	0.3 μM
GVR-EB-Mpx-rp	GCAACGCGAAGAACCTTACCT			
	**7**. ***L. monocytogenes***			
GVR-LM-Mpx-up	TGACGAAATGGCTTACAGTGA	*hly*	163	1 μM
GVR-LM-Mpx-rp	GCCGAAGTTTACATTCAAGCT	(listeriolysin O)		
	**8**. ***Salmonella*** **spp**.			
GVR-SE-Mpx-up	CCCGATTTTCTCTGGATGGT	*invA*	176	0.5 μM
GVR-SE-Mpx-rp	GGCAATAGCGTCACCTTTGA	(invasion gene at the pathogenicity island 1)		
	**9**. ***St. aureus***			
GVR-SA-Mpx-up	GCAACTGAAACAACAGAAGCT	*coa*	101	0.5 μM
GVR-SA-Mpx-rp	TCACGGATACCTGTACCAGCA	(staphylocoagulase)		

The genome size and mass for a GE of each pathogen (used as reference for evaluating detection limits) are: 5.4 Mb and 0.0055214 pg for *B. cereus*, 1.64 Mb and 0.0016784 pg for *C. jejuni*, 3.3 Mb and 0.0033742 pg for *Cl. perfringens*, 4.51 Mb and 0.0046114 pg for *Cr. sakazakii*, 5.6 Mb and 0.0057259 pg for *E. coli* and for *Enterobacteriaceae*, 2.85 Mb and 0.0029141 pg for *L. monocytogenes*, 2.9 Mb and 0.0029652 pg for *St. aureus*, and 4.6 Mb and 0.0047034 pg for *Salmonella* spp. With respect to *Enterobacteriaceae* family, the used reference GE size was the one corresponding to *E. coli*.

### DNA electrophoresis

Capillary electrophoresis of mPCR reactions products were performed using an Agilent 2100 Bioanalyzer® with DNA 1000® chips. These results were analyzed with 2100 Expert Software (Agilent), following the instructions provided by the manufacturer.

### Sequencing

Amplicons, obtained by mPCR, from each target gene were sequenced on the Sequencing Core Facility at the Universidad de Oviedo, using an ABI PRISM 3130 XL Genetic Analyzer (Life Technologies). Results were analyzed with BioEdit Sequence Alignment Editor v. 7.0.5.3 (Hall, 1999). Target sequences alignment was performed using the Clustal W web tool (Larkin et al., 2007).

### External evaluation

The developed method was externally evaluated using five different groups of food matrices as follows: meat products (grounded bovine meat), dairy products (powder milk), prepared food (canned cooked beans), fish products (canned fish), and pastry (sponge cake). A control without food matrix was included.

Ten replicates of 10 g for each one of these food matrices (or control without matrix) were mixed with GVRUM medium up to 100 g final weight in a stomacher bag. Spiking was carried out inoculating with 300 CFUs/mL of each pathogen individually in each bag and incubating it at 37°C during 18 h.

Two 1 mL samples from each one of the 540 bags were obtained. One of these samples was used for carrying out serial dilutions in GVRUM medium until 10^−9^ and plating 100 μL (duplicate experiments) of these on the corresponding growth medium for the tested pathogen (see Table 2). The second 1 mL sample was used for extracting gDNA as it has been described before.

## Results

### Design of a universal pre-enrichment medium (GVUM)

In order to allow growth of those pathogens with more specific nutritional requirements, GVUM was designed using a base made of BPW (20 g/L), where some extra components were sequentially added along its developing process. Specifically, mannitol was included in this formula (20 g/L) in order to help growth of *St. aureus*, as this bacterium does not show a good growth rate in just BPW. Conventional media for this species, MSA (Mannitol Salt Agar), contain this sugar (as well as diverse hydrolyzed proteins) as the main energy substrate (Cominazzini and Cavallina, 1961).

At a second improvement step, in order to facilitate growth of *C. jejuni*, hemolyzed and defibrinated horse blood was included in the composition of GVUM medium (at 50 mL/L). Current *C. jejuni* pre-enrichment media contain this lysed blood supplement (as in Columbia Blood- or Bolton-based Media) (Kunze, 1971; Bolton and Coates, 1983) as a way to quench oxygen because this bacterium requires microaerophilic conditions (less than 6% oxygen). Conventional *C. jejuni* media also contain a mixture of ferrous sulfate, sodium metabisulfite, and sodium pyruvate, as extra ingredients to increase aero-tolerance and to protect cells from damage (McNemar, 1947). GVUM contains these three ingredients from commercial *Campylobacter* Growth Supplement, added at 4 mL/L. The use of hemolyzed and defibrinated horse blood as ingredient in GVUM is essential to allow cell density measurement at 600 nm, as with regular blood the presence of intact erythrocytes impairs this 600 nm measurement.

These modifications in BPW composition were able to allow growth and detection of all nine types of pathogens in GVUM. This was true also in the case of *Cl. perfringens*, an anaerobic species that probably takes advantage of local anaerobic environments created in the mixture of GVUM with the different tested food matrices, but also clearly benefits from the presence of an array of oxygen quenching components in the GVUM formula. Also, all types of *Enterobacteriaceae* and all species belonging to *Salmonella* genus were able to grow individually in this GVUM medium.

Moreover, using this new developed GVUM medium, together with each one of the different 19 types of food matrices (and control bags without matrix, just GVUM), excellent growth of each individual bacterial types was achieved (*B. cereus, C. jejuni, Cl. perfringens, Cr. sakazakii, E. coli*, bacteria form the *Enterobacteriaceae* family, *L. monocytogenes, St. aureus*, and *Salmonella* spp.). These 19 types of food matrices correspond to the ones defined in the NordVal Validation Protocol: bovine grounded meat, fresh chicken, fresh fish, canned fish, fish *consommé*, fresh and cured sausages, raw milk, yogurt, two different infant milk formulae (with and without plant starches), cheese, cream, raw and cooked eggs, pastry, honey, dairy dessert, and vegetables. Experiments for quantifying these growths were carried out three times using one representative species of each group. These CFUs numbers are shown in Table 2. These colonies were confirmed for each one of the pre-enriched pathogens by using serial dilutions on the corresponding selective and differential media, observing an exponential increase of colonies about initial inocula.

Also, in order to evaluate how efficient was the enrichment of each individual species in a mixed culture in GVUM, a representative of each one of the nine bacterial types was inoculated (by triplicate) together with other eight species in a GVUM bag, as described in previous section (300 CFU/g of medium). In these experiments, *E. coli* was included also as representative of *Enterobacteriaceae* family. After 18 h pre-enrichment at 37°C, 10 decimal serial dilutions were made (using GVUM medium), and 100 μl were spread on the corresponding selective agar medium plates (see Materials and Methods Section), and incubated for 24 h. The resulting isolated colonies, at the corresponding dilution, were counted and an average CFUs number obtained from each three-replicate experiment (Table 2). These data demonstrate that, when incubating at the same time all different types of bacteria in GVUM, no overgrowth of one species against other ones takes place. This is very important as otherwise; some bacterial species could impede the normal pre-enrichment of other ones, invalidating the final objective of the experiment.

Finally, in order to evaluate if some competition between bacterial species could take place during enrichment in GVUM medium, we carried out two different enrichment experiments (duplicate experiments each one) by inoculating 10 CFUs/g (total 1000 CFUs/100 g GVUM batch) of *Salmonella* sp. (as example of *Enterobacteriaceae*) plus 1000 CFUs/g (total 100,000 CFUs/100 g batch) of the other seven microorganisms. A similar experiment was carried out in parallel with 10 CFUs/g (total 1000 CFUs/100 g GVUM batch) of *L. monocytogenes* (as an example of non-*Enterobacteriaceae*) plus 1000 CFUs/g (total 100,000 CFUs/100 g batch) of the other seven microorganisms. Results are shown in Table 2 and these ones demonstrate that there is not strong competition in this universal pre-enrichment medium (GVUM), as *Salmonella* and *L. monocytogenes* can achieve high CFUs (although slightly lower than in control batches) in both experiments (Table 2).

### Selection of the specific gene target for each pathogen

In order to ensure that the developed mPCR detection method would be suitable for commercial purposes targeting the nine described pathogens, a careful selection of gene targets for each one of these pathogens was carried out. The genes targets were selected on the basis of being highly specific of family, genus, species or strain, depending on the specific case. Genes coding for strain-specific variants of toxins (as some enterotoxins) were discarded in order to avoid false negatives. The reason to include 13 *Salmonella* strains was the high genetic and immunologic variability in this pathogen. In the case of the Family *Enterobacteriaceae*, a meticulous selection of all possible genera able to grow in food matrices (pathogenic and harmless) was carried out. With all these constrictions, the finally selected gene targets were those ones described in Table 3.

### Design of oligonucleotide pairs for each targeted gene

**Figure 1:**
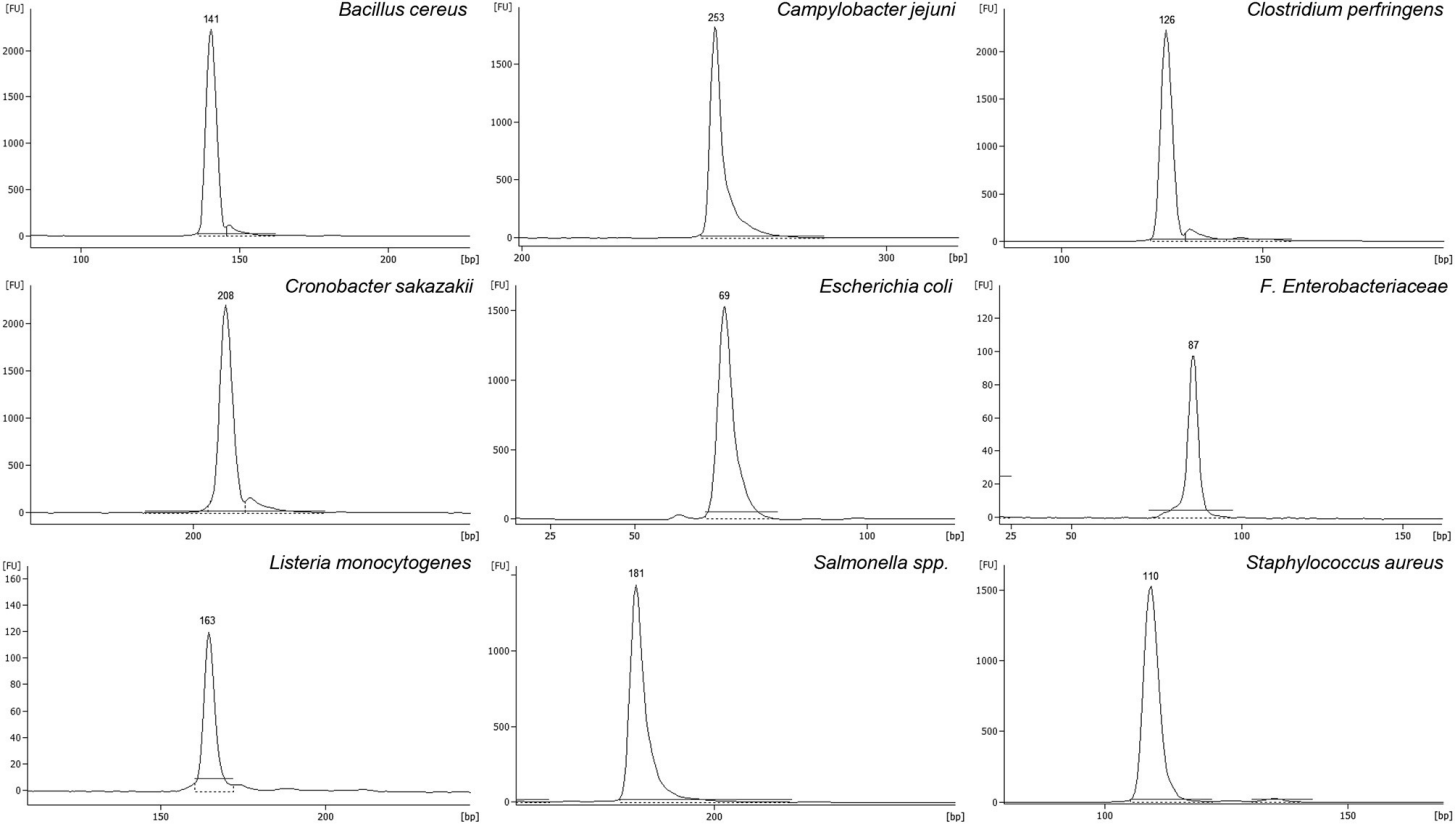
**Amplicons obtained by mPCR with primer pairs designed for: ***E. coli*** (EC), F. ***Enterobacteriaceae*** (EB), ***St. aureus*** (SA), ***Cl. perfringens*** (CP), ***B. cereus*** (BC), ***L. monocytogenes*** (LM), ***Salmonella*** spp. (SE), ***Cr. sakazakii*** (CS), and ***C. jejuni*** (CJ)**. Values showed on peak's top, corresponds to the size detected for each amplicon in bp.

Different oligonucleotide pairs were designed for each pathogen, until achieving total optimization and performance. All of them were individually tested against pure gDNA from the corresponding pathogen in a monoplex PCR, visualizing the expected amplicon in capillary electrophoresis (Figure 1). From these gels, each amplicon was isolated and sequenced, demonstrating that the amplified region of the pathogen gDNA was the expected one. Also, each oligonucleotide pair was tested against the other pathogens and against the negative control species, in order to test that no cross amplification (false positives) was taking place. The list of negative control species is listed in Table 1. Positive amplification was obtained always only in presence of the specific gene target, and never when using negative control strains. Also, in order to test the effect of possible inhibitors from food matrices, PCR experiments were carried out with gDNA from the different spikings described in Section Food Samples Spiking using 19 types of food matrices. In all cases, positive PCR amplification was obtained for each microorganism, independently of the food matrix used, and demonstrating that this method is not affected for possible inhibitors deriving from any of these 19 types of food matrices (Figure S1).

The final versions of these oligonucleotides, as well as the size of each one of the nine amplicons are shown in Table 3.

### mPCR detection method optimization for the nine pathogens

**Figure 2:**
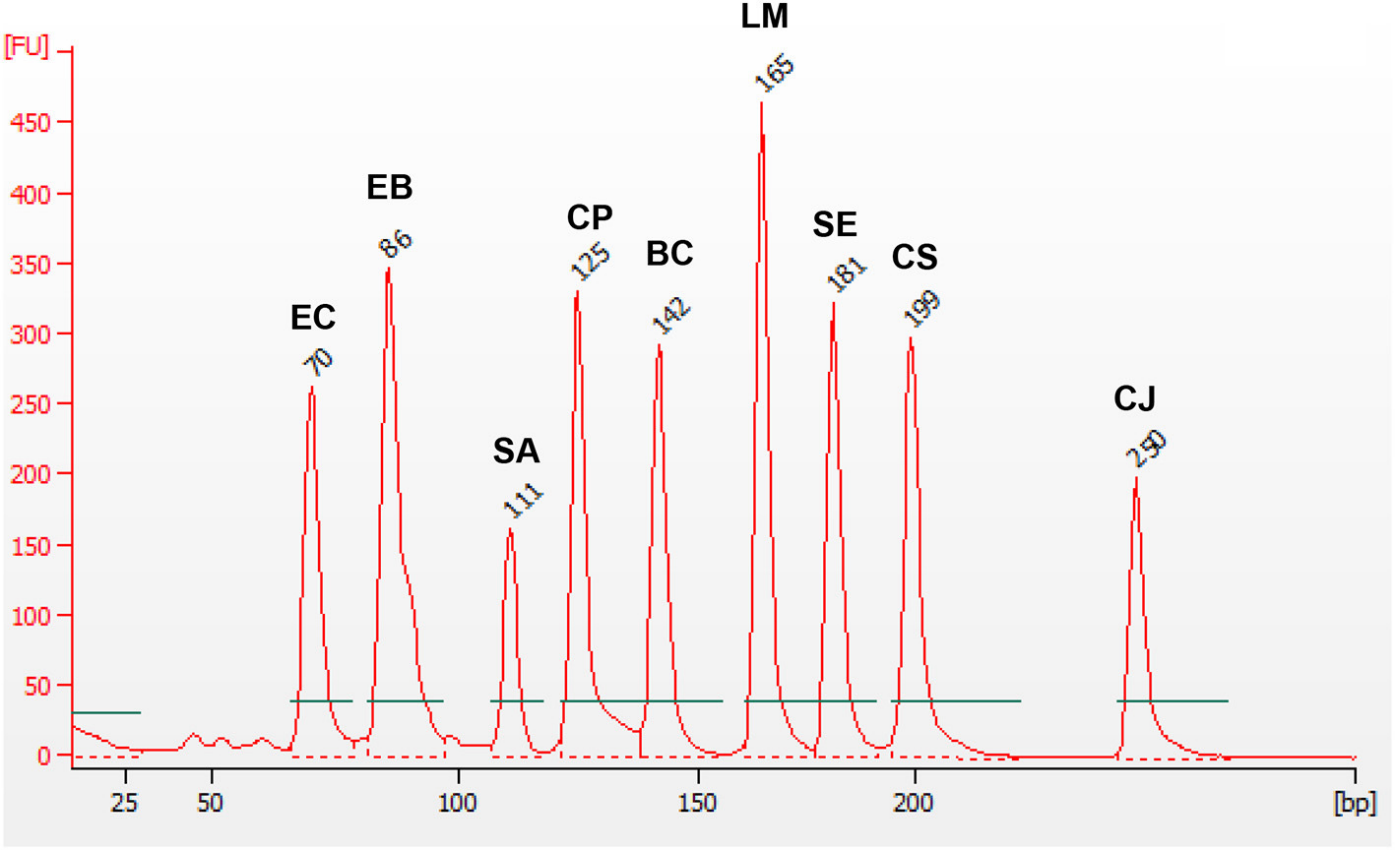
**mPCR with 9 pairs of primers and gDNA of ***B. cereus*** (BC), ***C. jejuni*** (CJ), ***Cl. perfringens*** (CP), ***Cr. sakazakii*** (CS), ***E. coli*** (EC), ***F. Enterobacteriaceae*** (EB), ***L. monocytogenes*** (LM), ***Salmonella*** spp. (SE), and ***St. aureus*** (SA), at ***Tm*** = 56°C**. The values showed on each peak, corresponds to the size detected for each amplicon.

As it has been described in Materials and Methods Section, optimization of the 9-plex PCR was carried out starting with the PCR optimization for each individual pathogen at 16 different conditions of oligonucleotides concentrations and amplification temperatures. Then, a PCR optimization with two pathogen species, again at several amplification conditions, was carried out. The optimization process was started using the primers designed for the genus *Salmonella*. Once positive amplification was obtained and the optimal concentration (1 μM for each primer) and Tm (56°C) was established, the optimization continues adding the couple of primers designed for *St. aureus*. This process was repeated sequentially until inclusion of all nine pathogen types (see Materials and Methods Section). Finally, positive amplification for all nine targets was obtained (see Figure 2).

One bag filled only with 100 g of GVUM was included as the negative control for all experiments, in order to show possible interference caused by food matrices. These bags were spiked with the corresponding pathogens. In all cases, the growth was observed and the mPCR reaction showed positive amplifications. These experiments were not repeated during the evaluation step.

**Figure 3:**
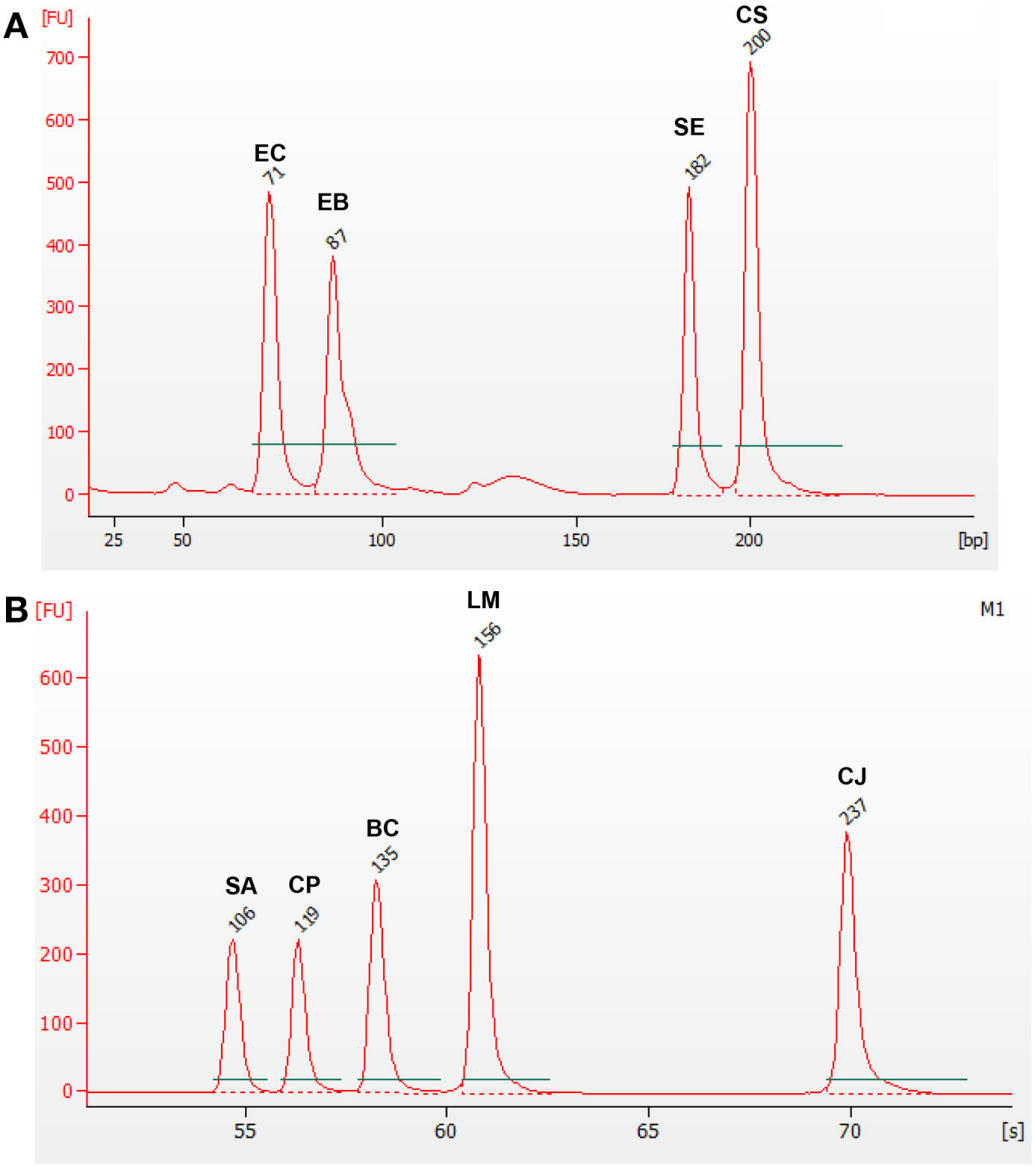
**mPCR using gDNA from two groups of microorganisms**. Electropherogram **(A)** corresponds to *Enterobacteriaceae* microorganisms*: Cr. sakazakii* (CS)*, E. coli* (EC), *Salmonella* spp. (SE), and F. *Enterobacteriaceae* (EB); while electropherogram **(B)** corresponds to the non-*Enterobacteriaceae* microorganisms: *B. cereus* (BC)*, C. jejuni* (CJ)*, Cl. perfringens* (CP), *L. monocytogenes* (LM), and *St. aureus* (SA).

Additionally, two other mPCR reactions were designed, in order to amplify two subgroups of pathogens, those belonging to the *Enterobacteriaceae* family (*Cr. sakazakii, E. coli*, and *Salmonella* spp.) and the other species (*B. cereus, C. jejuni, Cl. perfringens, L. monocytogenes*, and *St. aureus*). This is of interest for some food sectors, and it is also a proof of the versatility of the designed detection system. In these two other mPCR, the gDNAs for the corresponding subgroups of pathogens were tested against all nine oligonucleotide pairs (Figure 3).

Finally, once all mPCR conditions were fixed, PCR amplifications were carried out, using four replicates for each pathogen, taking as template serial dilutions of pure gDNA of the corresponding pathogen, previously quantified by spectrophotometry. These experiments allowed determination of the minimum detection threshold for each pathogen, as gDNA template concentrations were calculated in terms of genome equivalents (GE) for the corresponding pathogen, using the formula: Genome (pb) = mass (pg) • 0.978 • 10^9^. Using this formula it is possible to determine the mass of one genome for each type of pathogen, as well as to convert into GE the gDNA mass used in these PCR experiments. In all cases, it was possible to get positive PCR amplification signals from these pathogens in the assayed dilutions of gDNA. These experiments were able to detect 1 GE for all pathogens, in all cases (four replicates each). But in the case of the family *Enterobacteriaceae*, these were able to detect 5 GE.

For each target pathogen, three other species were used as negative controls, and selected and tested with the corresponding mPCR primer pairs. In all cases, negative control species produced no PCR amplification.

### Evaluation of the mPCR against traditional culture detection methods

In order to test the reproducibility and performance of the designed mPCR detection method for the nine pathogens, the developed mPCR was compared with the traditional detection method based on cultivation of serial dilutions from the pre-enrichment medium. gDNA samples from these pre-enrichment experiments were used for mPCR reactions (duplicate experiments). The presence of colonies in these media (absence/presence) and the mPCR results of all these raw experiments are shown in Table 4. The raw data were processed and used to construct a contingency table to determine the percentage of coincidence between classical and mPCR detection methods, and to perform a McNemar's statistical test (McNemar, 1947) to validate the equality between these two detection methods (Table 5). This test is based in the (*H*_0_) null hypothesis of equality on discordant data pairs between both evaluated methods.

**Table 4:** **Results obtained after detection, using classical microbiology (M) and mPCR (PCR) methods**.

	***B. cereus***		***C. jenuni***		***Cl. perfringens***		***Cr. sakazakii***		***E. coli***		***Enterobacteriaceae***		***L. monocytogenes***		***St. aureus***		***Salmonella*** **spp**.	
**Sample**	**M**	**P**	**M**	**P**	**M**	**P**	**M**	**P**	**M**	**P**	**M**	**P**	**M**	**P**	**M**	**P**	**M**	**P**
		**C**		**C**		**C**		**C**		**C**		**C**		**C**		**C**		**C**
		**R**		**R**		**R**		**R**		**R**		**R**		**R**		**R**		**R**
**MEAT PRODUCTS**																		
1	P	P	P	P	A	P	P	P	P	P	P	P	A	P	P	P	P	P
2	P	P	P	P	P	P	A	P	P	P	P	P	P	P	P	P	P	P
3	P	P	P	A	P	P	P	P	P	P	P	P	P	P	P	P	P	P
4	P	P	A	P	P	A	P	P	P	P	P	P	P	P	P	P	P	A
5	P	P	P	P	P	P	P	A	A	P	P	P	P	P	P	P	P	P
6	P	P	A	A	P	P	P	P	P	P	P	A	P	A	P	A	P	P
7	P	P	P	P	P	P	P	P	P	P	A	P	P	P	P	P	P	P
8	P	P	P	P	P	P	P	P	P	P	P	P	P	P	P	P	P	P
9	P	P	P	P	P	P	A	P	P	P	P	P	P	P	P	P	P	P
10	P	P	P	P	A	P	P	P	P	A	P	P	P	P	P	P	P	P
**PREPARED FOOD**																		
1	P	P	P	P	P	P	P	P	P	P	P	A	P	P	A	A	P	P
2	P	P	P	A	P	P	P	P	P	P	A	P	P	P	P	P	P	P
3	P	P	P	P	P	P	P	P	P	P	P	P	P	P	P	P	A	P
4	P	P	P	P	A	A	P	P	P	P	P	P	P	P	P	P	P	P
5	P	P	P	P	P	P	P	P	P	A	P	P	P	P	P	P	P	P
6	P	P	P	A	P	P	P	P	P	A	A	P	P	P	P	P	P	P
7	P	P	P	P	P	P	P	P	P	P	P	A	P	P	P	P	P	P
8	P	P	P	P	A	P	P	P	P	P	P	P	P	A	P	P	A	P
9	P	P	P	P	P	P	A	P	P	P	P	P	P	P	A	P	P	P
10	P	P	P	P	P	P	P	P	P	P	P	P	P	P	A	A	P	P
**FISH PRODUCTS**																		
1	P	P	P	P	P	P	P	P	P	P	P	P	P	P	P	P	P	P
2	P	P	A	P	P	P	P	P	P	P	P	P	P	P	A	P	P	P
3	P	P	P	A	A	P	P	P	P	P	P	P	P	A	P	P	P	P
4	P	P	P	P	P	P	P	P	A	P	P	P	P	P	P	P	P	A
5	P	P	P	P	P	P	P	P	P	P	P	P	P	P	P	P	P	P
6	P	P	A	P	A	A	P	P	A	A	P	P	P	P	P	A	P	P
7	P	P	P	P	P	P	P	P	P	P	P	A	P	P	P	A	P	P
8	P	P	P	P	P	P	P	P	P	A	P	P	P	A	A	P	P	P
9	P	P	P	P	P	A	P	P	P	P	P	P	P	P	P	P	P	P
10	P	P	P	P	P	P	P	P	P	P	P	P	P	P	P	A	P	P
**PASTRY PRODUCTS**																		
1	P	P	P	P	P	P	P	P	P	P	P	P	P	P	P	A	P	P
2	A	P	P	P	P	P	P	P	P	P	P	P	P	P	P	P	P	P
3	P	P	P	P	P	P	P	P	P	P	P	P	P	P	P	P	P	P
4	P	P	P	P	A	P	P	P	P	P	P	P	P	P	P	A	P	A
5	A	P	P	P	P	A	A	A	P	P	A	A	P	P	P	P	P	P
6	P	P	P	P	P	P	P	P	P	P	P	P	A	P	P	P	P	P
7	P	A	A	A	P	P	P	P	P	A	P	P	P	A	P	P	P	A
8	A	P	P	P	P	P	P	A	P	P	P	P	P	P	P	P	A	P
9	P	P	P	P	P	P	P	P	P	P	P	P	P	P	P	P	P	P
10	P	P	A	P	A	P	P	P	P	A	A	A	P	P	A	P	P	P
**DAIRY PRODUCTS**																		
1	P	P	P	P	P	P	P	P	P	P	P	P	P	P	P	P	P	P
2	P	P	P	P	P	P	P	P	P	P	P	P	A	P	P	A	P	P
3	P	P	P	A	P	P	P	A	P	P	P	P	A	P	P	P	A	P
4	P	P	P	P	P	A	P	P	P	P	P	P	P	P	P	P	A	P
5	P	P	P	P	A	P	P	P	P	P	A	P	P	P	P	P	P	P
6	P	P	A	A	P	P	P	P	P	P	P	P	P	P	P	P	P	P
7	P	P	P	P	P	P	P	P	P	P	P	P	P	P	P	P	P	P
8	P	P	P	P	P	P	A	P	P	P	P	P	P	P	P	P	P	A
9	P	P	A	A	P	P	P	P	P	P	P	P	P	A	P	P	P	P
10	P	P	P	P	P	P	P	A	P	P	P	P	P	P	P	P	P	P
**SUMMARY**																		
PP	46		37		37		41		41		40		40		37		40	
AA	0		4		2		1		1		2		0		2		0	
PA	1		5		4		4		6		4		6		7		5	
AP	3		4		7		4		2		4		4		4		5	

*P, Presence; A, Absence; PP, Presence/Presence by microbiological and mPCR method, respectively; AA, Absence/Absence by microbiological and mPCR method, respectively; PA, Presence/Absence by microbiological and mPCR method, respectively; AP, Absence/Presence by microbiological and mPCR methods, respectively*.

**Table 5:** **General contingency tables for nine microorganisms, showing percentage of coincidence and ***p***-value for McNemar's test**.

		**mPCR**			**% Coincid**.	**McNemar *p* val**.
		**Absence**	**Presence**	**Total**		
***B. cereus***						
**Classic**	Absence	0	3	3	92%	0.6171
	Presence	1	46	47		
	Total	1	49	50		
***C. jejuni***						
**Classic**	Absence	4	4	8	82%	0.7237
	Presence	5	37	42		
	Total	9	41	50		
***C. perfringens***						
**Classic**	Absence	2	7	9	78%	0.5465
	Presence	4	37	41		
	Total	6	44	50		
***C. sakazakii***						
**Classic**	Absence	1	4	5	84%	0.7237
	Presence	4	41	45		
	Total	5	45	50		
***E. coli***						
**Classic**	Absence	1	2	3	84%	0.2888
	Presence	6	41	47		
	Total	7	43	50		
***Enterobacteriaceae***						
**Classic**	Absence	2	4	6	84%	0.7237
	Presence	4	40	44		
	Total	6	44	50		
***L. monocytogenes***						
**Classic**	Absence	0	4	4	80%	0.7518
	Presence	6	40	46		
	Total	6	44	50		
***S. aureus***.						
**Classic**	Absence	2	4	6	78%	0.5465
	Presence	7	37	44		
	Total	9	41	50		
***Salmonella*** **spp**.						
**Classic**	Absence	0	5	5	80%	0.7518
	Presence	5	40	45		
	Total	5	45	50		

*M, microbiological method; PCR, mPCR method*.

As result from these comparisons, coincidence percentages were very high for all pathogens, starting at 78% in the case of *Cl. perfringens*, and reaching the highest value of 92% for *B. cereus*. Also, McNemar's tests showed *p* > 0.05 (Table 5), which indicate no statistical significance of this data. Therefore, the H0 null hypothesis was accepted, thus confirming the equivalence between both methods to detect this set of nine microorganisms.

## Discussion

Some of the pathogens used in this work (as *B. cereus, Cl. perfringens, E. coli*, total enterobacteria, and *St. aureus*), are usually present in some food matrices in such high quantities (over thousands per matrix gram) that it would be really complicate to avoid its detection due to technical errors during plating. Also, the allowed limits for these pathogens under most food legislations are hundreds or thousands of CFUs/g, as minor amounts do not represent a serious health risk for the general consumer.

However, the detection of other more dangerous pathogens (*C. jejuni, Cr. sakazakii, E. coli* O157:H7, *L. monocytogenes*, and *Salmonella* spp.), is a very important issue. In these cases, a pre-enrichment step is mandatory, in order to ensure that even the presence of few cells in the analyzed food matrix is going to be detected. During this pre-enrichment step, those putative low CFUs will multiply to high numbers. This fact facilitates the later detection of those bacteria, avoiding false negatives.

Current standard pre-enrichment methods recommend the use of BPW medium, which contains peptone (an enzymatic hydrolysis product from animal proteins) (10 g/L) as energy and biomolecules source (including required trace elements), sodium chloride (5 g/L), disodium phosphate (3.5 g/L), and potassium dihydrogen phosphate (1.5 g/L). These last two components maintain the initial buffered pH at 7.2, once the corresponding food matrix has been added. Using this medium, most pathogenic bacterium can be pre-enriched from food matrices, including those cells injured during food manufacturing and/or preservation processes. Its buffered condition also avoids a drop in medium pH when some special food matrices (as some vegetables) are used during experimentation. The low pH values achieved in these cases could also inhibit some bacterial pathogens as *Salmonella*, something that is prevented by using this buffered medium (Hussein and Bollinger, 2008; Sánchez et al., 2012).

Although most food-borne bacterial pathogens are pre-enriched in BPW, some important ones are not able to grow in this medium, due to special nutritional or environmental requirements. This is the case of *C. jejuni* and *Cl. perfringens*, which are usually pre-enriched in Bolton Broth (BB) and Reinforced Clostridium Medium (RCM), respectively. BB contains, apart from peptone and other nutrients, haemin, sodium pyruvate and sodium metabisulfite. These components are in charge of lowering oxygen concentration, as this species is microaerophilic. On the other hand, RCM contains diverse sources of amino acids from protein hydrolysis, as well as cysteine hydrochloride in order to diminish final oxygen concentration.

To improve growth of most important food pathogens in artificially spiked food matrices, a universal pre-enrichment medium (GVUM) was developed in this work. This universal medium was able to create optimal conditions for growing all these microorganisms alone or together, and also in presence of 19 different food matrices, those from the NordVal validation system: bovine grounded meat, fresh chicken, fresh fish, canned fish, fish *consommé*, fresh and cured sausages, raw milk, yogurt, two different infant milk formulae (with and without plant starches), cheese, cream, raw and cooked eggs, pastry, honey, dairy dessert, and vegetables. This was demonstrated by plating cultures of each pathogen from GVUM on the canonical selective medium for that species, obtaining positive growth confirmation in all cases. GVUM is, therefore, very useful in order to simplify routine praxis in laboratories dealing with analyses for microbial quality of different food types, as using just a single pre-enrichment step during spiking, allows the analysis of diverse pathogens. In contrast, current methods require the use of different pre-enrichment media depending on each pathogen species. This fact increases the amount of time for media preparation.

Due to their worldwide incidence, food-borne microbial pathogens have a major importance in the economy and health. In food industry and diagnosis laboratories, the most common methods used to detect these microorganisms are those based in classical microbiology techniques. These methods are tedious as well as demonstrate poor accuracy (in the absence of selective enrichment), and also do not allow fast detection for a high number of different pathogens. DNA-based methods, like PCR, enable an important time reduction for getting results, saving money in manufacturing plants and reducing response time to clinical outbreaks. Different PCR-based detection methods for some food-borne pathogens have been developed in the last years (Alarcón et al., 2004), and some of these methods have been coupled to automatic detection systems of the corresponding PCR amplification bands by using capillary electrophoresis (Carli et al., 2001).

However, in this work we have achieved the simultaneous detection for nine types of food-borne pathogens (seven species, one genus and one family of food-borne pathogens), using just a single type of universal pre-enrichment method, and detecting the amplification signals by a cheap and easy-to-use capillary electrophoresis apparatus commonly used at clinical and industrial level. As shown in Figure S1, these detections can also be performed by agarose gel electrophoresis, but the sensitivity is much lower than with capillary electrophoresis. This facilitates its future commercial development, although this method is qualitative, and in case of positive detection for some of these microorganisms (as for example *E. coli* and *Enterobacteriaceae*), further quantification or its microbial load would be necessary. For future development, an internal amplification control (IAC) could be included in order to avoid false negative results as a consequence of PCR inhibitors. Anyway, as shown in Figure S1, using 19 types of food matrixes (plus only GVUM as growth medium), absence of PCR amplification due to putative matrix inhibitors or experimental errors is very low, as from 265 PCR samples from these spiking experiments, only 1.88% shows no PCR bands (5 PCR reactions in total).

Nine gene targets have been selected for this, which are highly conserved in the genome of the corresponding pathogen in order to avoid false negatives in some strains or serovars. With respect to this, these experiments we carried out in the case of *Salmonella* spp. with 13 strains from two *Salmonella* species; and with 24 different genera of the *Enterobacteriaceae* family.

In order to avoid false positives, the selected gene targets are only present in the targeted pathogen or commensal bacteria, and not in close taxonomic relatives of the corresponding species. For this purpose, for each target pathogen or commensal bacteria, three other species as negative controls were selected and tested with the corresponding mPCR primer pairs. For example, in the case of *B. cereus* CECT-131 (pathogen), the negative control strains are *Bacillus badius* CECT-17T, *Bacillus pumilus* CECT-29T, and *Bacillus subtilis* CECT-35, which are considered non-pathogenic in humans.

The developed mPCR method presented here is sensitive and inexpensive for the nine most relevant bacterial food-borne pathogens and commensal bacteria. The method uses mPCR and capillary electrophoresis as fast detection technologies, and allows an important reduction in the required time for detection of these pathogens, as only 18 h (pre-enrichment) plus 2 h (mPCR) and 30 min (capillary electrophoresis) are required; in contrast with 18 h (pre-enrichment) plus 24–96 h (plating on solid media and confirmation of positives) as in traditional protocols. Culture-based methods also distinguish between viable and non-viable microorganisms. Ethidium Monoazide protocols can be used for discrimination of these aspects, when used coupled to the PCR detection protocol, as cross-linked EMA adducts (generated only from non-viable cells gDNA) are not suitable for PCR amplification.

Specific gene targets were selected for each microorganism and a set of nine oligonucleotide pairs were designed and tested individually, showing positive amplification only when the gene target was present on the sample, demonstrating the specificity between the oligonucleotides and their targets. The developed oligonucleotides in this work have been developed using consensus sequences from a great number of GenBank entries covering diverse sequenced isolates and strains for these nine types of food-borne bacteria. Amplification was never obtained when using negative control strains (three different ones were used for each targeted bacteria in this work, see Table 1 for the complete names list). In those reactions performed with non-specific gDNA, amplification was not observed, certifying the specificity between targets and oligonucleotides. However, some exceptions on this must be explained; the oligonucleotide pair designed for *Enterobacteriaceae* family was able to amplify gDNA from all 13 tested *Salmonella* strains, as well as *Cr. sakazakii, E. coli*, and the 24 *Enterobacteriaceae* genera members. The pair designed against *Salmonella* spp. was able to amplify the 13 strains (belonging to two species) present in the collection.

A mPCR reaction was optimized, being able to amplify the nine gene targets simultaneously. During the optimization process for this 9-plex PCR reaction, differences between theoretical and experimental amplicon sizes were observed. These differences can be explained due to the accuracy and the resolution of the DNA100 Chip used to perform capillary electrophoresis on Agilent 2100 Bioanalyzer. These DNA chips have a resolution of ± 5 bp on amplicons up to 500 bp of length and an accuracy of 10% between different runs, so some variability was expected. Due to this experimental error for the capillary electrophoresis system, the following variation % for these amplicons, as detected in the Agilent 2100 Bioanalyzer were: *B. cereus* + 7.0%; *C. jejuni* ± 6.3%; *Cl. perfringens* + 7.9%; *Cr. sakazakii* + 5.7%; *E. coli* ± 1.4%; Fam. *Enterobacteriaceae* + 11.4%; *L. monocytogenes* ± 4.2%; *Salmonella* spp. + 3.2%; *St. aureus* + 9.9%. These percentages in between different runs of capillary electrophoresis are in accordance with the manufacturer. However, these differences were constant for each target during different experiments, allowing to unequivocally associate peaks to gene targets. Also, in all cases, different experiments always showed the same position (size) of the corresponding amplicon in the electropherogram. mPCR was carried out also with just each gDNA individually, in order to test reproducibility and specificity of the designed conditions. These individual amplicons were sequenced, and the obtained sequence was always the expected one. The same amplification and detection results were obtained when using a collection of 44 different strains of these bacteria from food and clinical isolates, which demonstrates that the designed oligonucleotides are broadly useful for diverse strains of the different food-borne bacteria analyzed in this work (Figure S2).

This 9-plex PCR detection method was externally evaluated against the classical method, using five types of food matrices. The equality between mPCR and classical methods was confirmed by a high coincidence percentages (>70%) and statistically non-significant *p*-values for McNemar's tests.

These results demonstrate that the proposed mPCR detection method is accurate and can be carried out in a shorter time (18 h pre-enrichment plus 2 h mPCR plus 30 min capillary electrophoresis) than current culture-dependent detection methods (18–24 h pre-enrichment plus 24–96 h cultivations). And, even more, this method does not require preparation of different pre-enrichment media depending on the bacterial species, as GVUM universal pre-enrichment medium works perfectly for any of the nine types of food pathogens/commensal bacteria, in any type of food matrix (19 types were used for each bacterial type, with excellent growth results).

Also, this mPCR method is much more inexpensive than other DNA-based detection methods, such as qRT-PCR or sequencing or microarrays. The calculated cost for each analysis encompassing nine types of bacteria, in any type of food matrix, and including mPCR and capillary electrophoresis steps, is about 2.75 €. These costs have been calculated using research lab consumables costs, not the expected lower costs when using these methods in high numbers as at industrial level. Also, the personnel costs with this method is much lower than with classical microbiology methods, as here there is no need for cultivation procedures after the pre-enrichment step, and all assays can be done at the same time for the nine types of pathogens or commensal bacteria.

### Conflict of interest statement

The authors declare that the research was conducted in the absence of any commercial or financial relationships that could be construed as a potential conflict of interest.

## Supplementary material

Figure S1 **Agarose gel electrophoresis for testing gDNA specific amplification from pre-enrichment cultures on GVUM, using 19 types of food matrixes contaminated with**. (1) *C. jejuni* (showing triplicate PCR experiments); (2) *E. coli*; (3) F. *Enterobacteriaceae* (in this case *K. pneumoniae*); (4) *St. aureus* (showing three replicates); (5) *Salmonella* spp. (in this case *Sa. enteriditis* CECT-556); (6) *L. monocytogenes*; (7) *B. cereus*; (8) *Cl. perfringens*; (9) *Cr. sakazakii*. CR, Fresh meat; P, Chicken; S, Sausages; F, Fish; CF, Canned fish; SF, Fish consommé; LC, Raw milk; Y, Yogurt; N, Cream; LP, Powder milk; Fi1, Infant formula 1; Fi2, Infant formula 2; HC, Eggs; V, Vegetables; M, Honey; W/O, GVUM control without matrix; C+, gDNA PCR positive control; C–, No template control.

Figure S2 **Amplicons obtained by mPCR for different strains of the tested bacterial species**. Values showed on peak's top corresponds to the size detected for each amplicon in bp. *B. cereus* strains: CECT-193, 83535, 34588, 53591, 83536, 83537, HUCA-431. *L. monocytogenes* strains: CECT-7467, 41319, 31925, 883, 103545-4, 61318, HUCA-17. *St. aureus* strains: CECT-435, 5083, 51555, 50698, 50700, 5070. *C. jejuni* strains: CECT-7572, 574-4, 574-5, 575-1, 575-2, 575-3, 575-4, 577-2, 577-4, 577-5, 664-1, 666-2, 1091-664031-VIII, 23151774331-VIII, 31-VIII, 15412091. *Cl. perfringens* strains: 41057, 31539, 194PMLS, 41226, 41887. *Cr. sakazakii* strains: CECT-858, 83562, 44179.
