# Supplementary material for: Multiplex detection of nine food-borne pathogens by mPCR and capillary electrophoresis after using a universal pre-enrichment medium
Source: Front Microbiol. 2015 Nov 3;6:1194. doi: 10.3389/fmicb.2015.01194 (PMC4630290; doi:10.3389/fmicb.2015.01194)
Supplement: Figure S1 — Agarose gel electrophoresis for testing gDNA specific amplification from pre-enrichment cultures on GVUM, using 19 types of food matrixes contaminated with. (1) C. jejuni (showing triplicate PCR experiments); (2) E. coli; (3) F. Enterobacteriaceae (in this case K. pneumoniae); (4) St. aureus (showing three replicates); (5) Salmonella spp. (in this case Sa. enteriditis CECT-556); (6) L. monocytogenes; (7) B. cereus; (8) Cl. perfringens; (9) Cr. sakazakii. CR, Fresh meat; P, Chicken; S, Sausages; F, Fish; CF, Canned fish; SF, Fish consommé; LC, Raw milk; Y, Yogurt; N, Cream; LP, Powder milk; Fi1, Infant formula 1; Fi2, Infant formula 2; HC, Eggs; V, Vegetables; M, Honey; W/O, GVUM control without matrix; C+, gDNA PCR positive control; C–, No template control. [file Presentation1.PDF]

Figure S1. Agarose gel electrophoresis for testing gDNA specific amplification from pre-enrichment cultures on GVUM, using 19 types of food matrixes contaminated with. 1) *C. jejuni* (showing triplicate PCR experiments); 2) *E. coli*; 3) F. *Enterobacteriaceae* (in this case *K. pneumoniae*); 4) *St. aureus* (showing three replicates); 5) *Salmonella spp*. (in this case *Sa. enteriditis* CECT-556); 6) *L. monocytogenes*; 7) *B. cereus*; 8) *Cl. perfringens*; 9) *Cr. sakazakii*. Legend: CR= Fresh meat; P=Chicken; S= Sausages; F= Fish; CF= Canned fish; SF= Fish consommé; LC= Raw milk; Y=Yogurt; N= Cream; LP= Powder milk; Fi1= Infant formula 1; Fi2= Infant formula 2; HC= Eggs; V= Vegetables; M= Honey; W/O= GVUM control without matrix; C+= gDNA PCR positive control; C-= No template control.

1

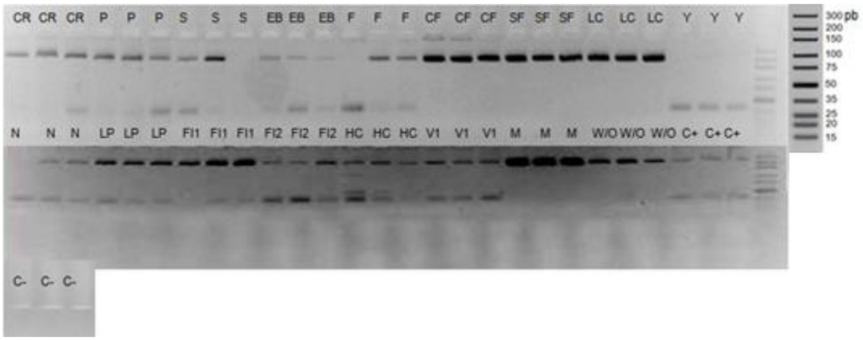

2

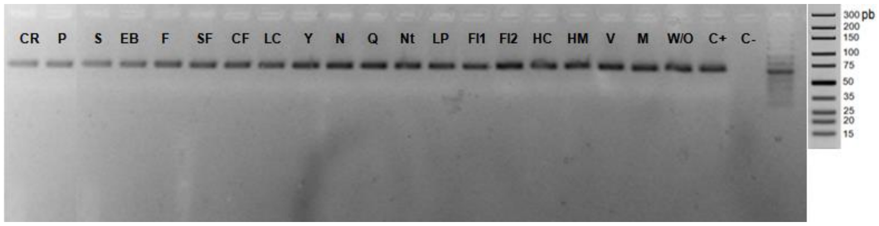

3

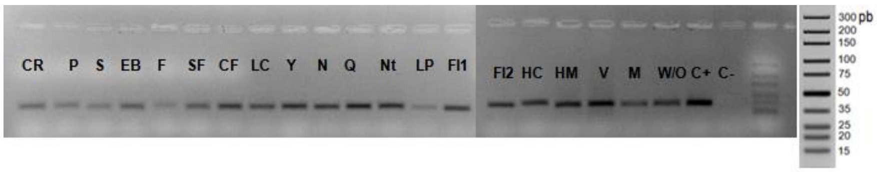

4

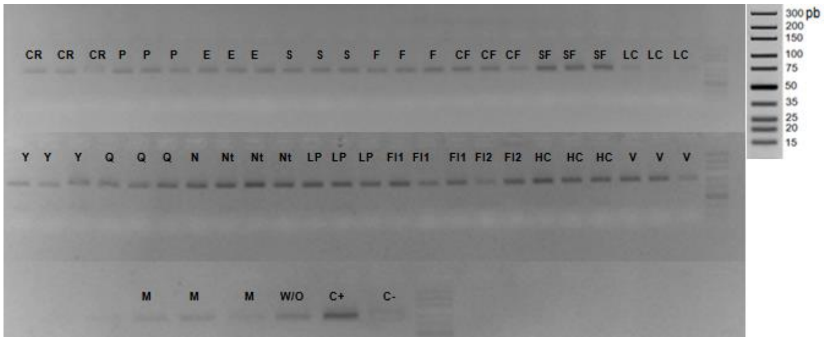
CR CR CR P P P E E E S S S F F F CF CF CF SF SF SF LC LC LC
Y Y Y Q Q Q N Nt Nt Nt LP LP LP FI1 FI1 FI1 FI2 FI2 HC HC HC V V V
M M M W/O C+ C-
300 pb 200 150 100 75 50 35 25 20 15

5

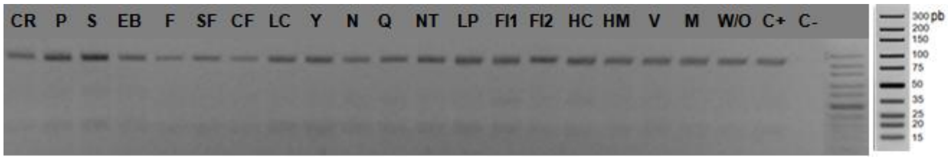
CR P S EB F SF CF LC Y N Q NT LP FI1 FI2 HC HM V M W/O C+ C-
300 pb 200 150 100 75 50 35 25 20 15

6

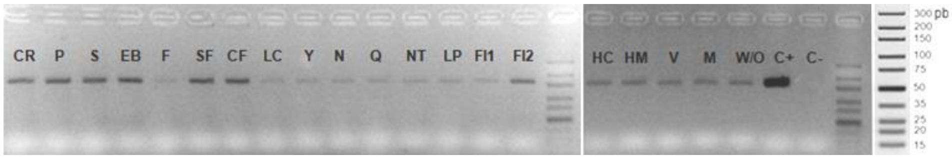
CR P S EB F SF CF LC Y N Q NT LP FI1 FI2
HC HM V M W/O C+ C-
300 pb 200 150 100 75 50 35 25 20 15

7

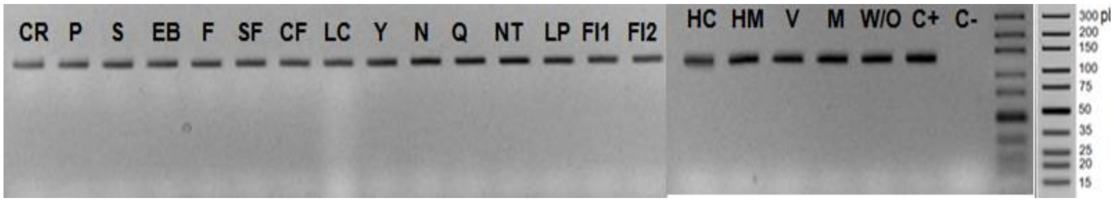
CR P S EB F SF CF LC Y N Q NT LP FI1 FI2
HC HM V M W/O C+ C-
300 pl 200 150 100 75 50 35 25 20 15

8

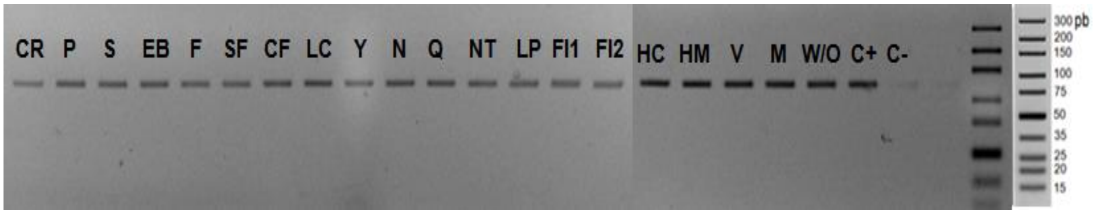
CR P S EB F SF CF LC Y N Q NT LP FI1 FI2
HC HM V M W/O C+ C-
300 pb 200 150 100 75 50 35 25 20 15

9

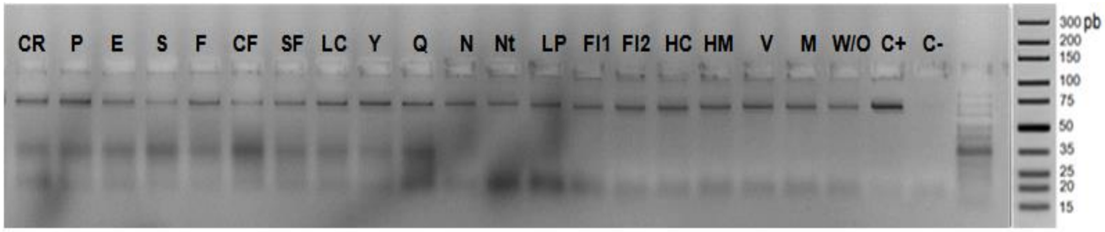
CR P E S F CF SF LC Y Q N Nt LP FI1 FI2 HC HM V M W/O C+ C-
300 pb 200 150 100 75 50 35 25 20 15
