# Supplementary figures and images for: Multiplex detection of nine food-borne pathogens by mPCR and capillary electrophoresis after using a universal pre-enrichment medium
Source: Front Microbiol. 2015 Nov 3;6:1194. doi: 10.3389/fmicb.2015.01194 (PMC4630290; doi:10.3389/fmicb.2015.01194)

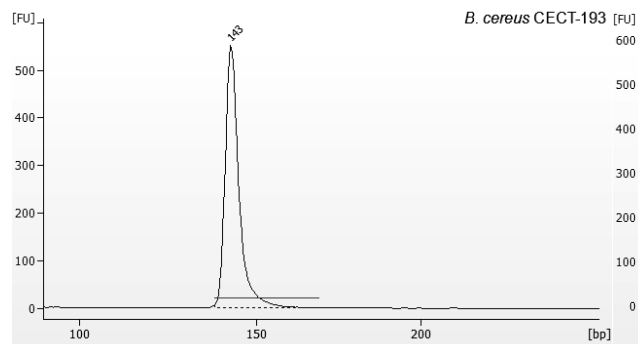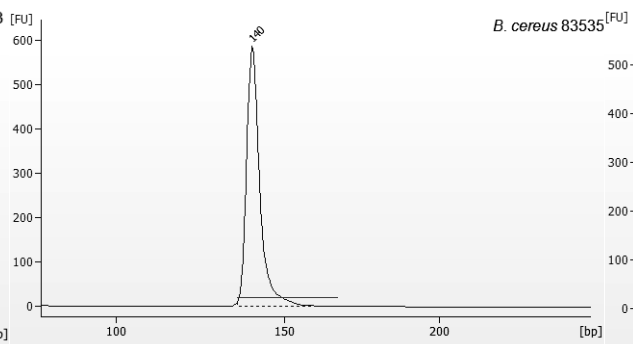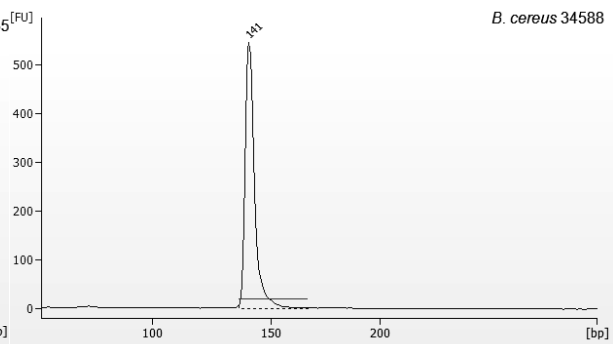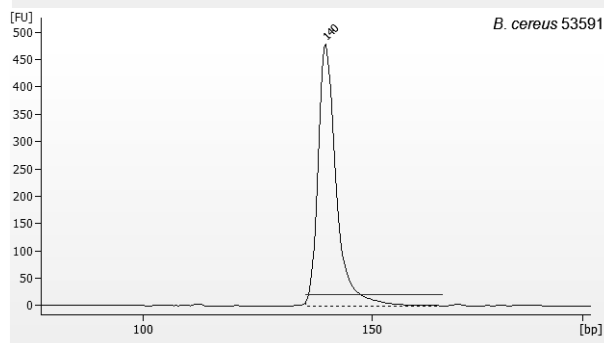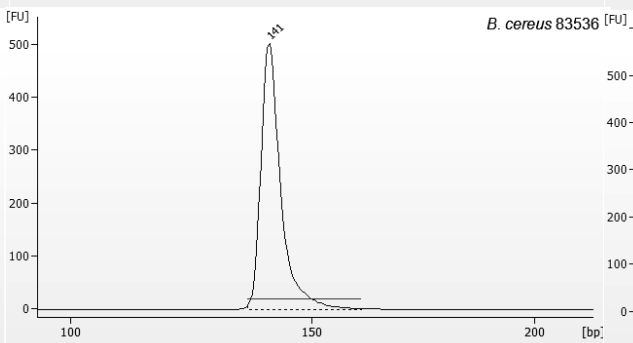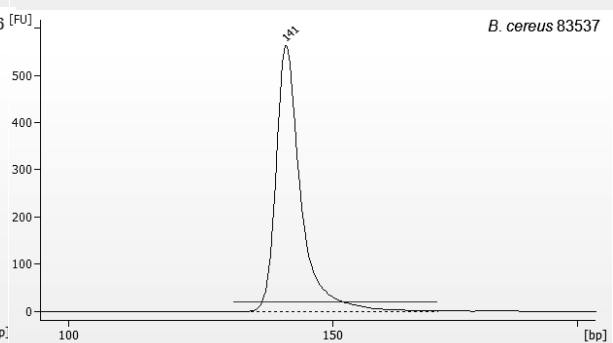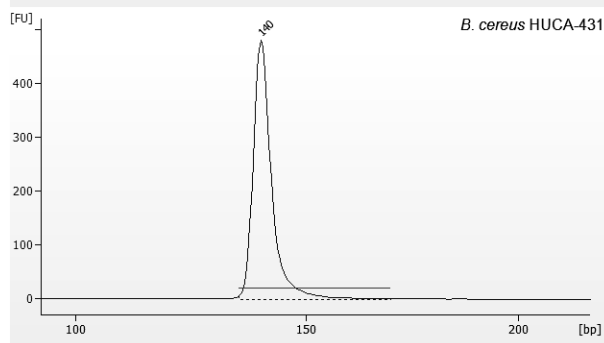

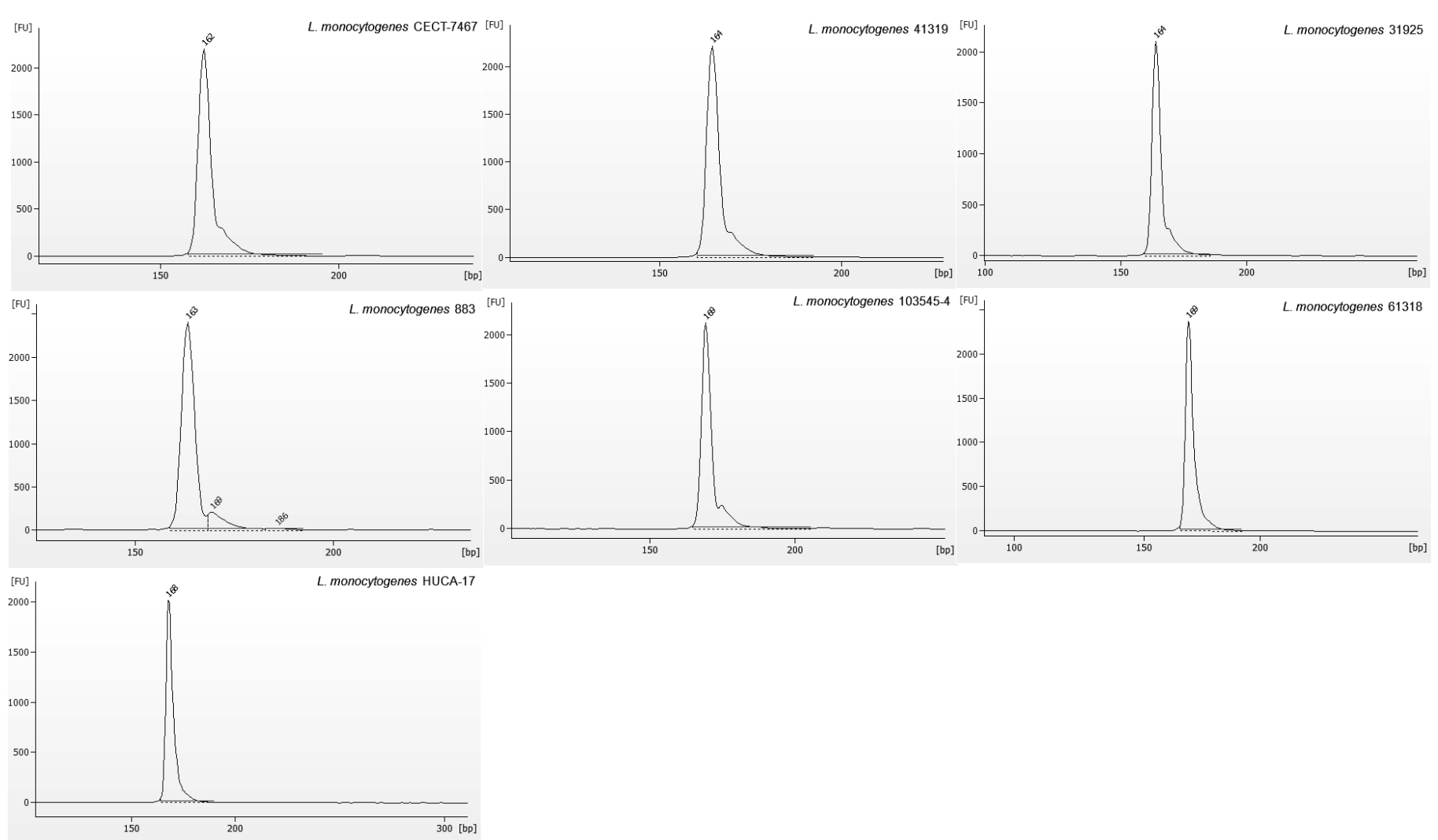

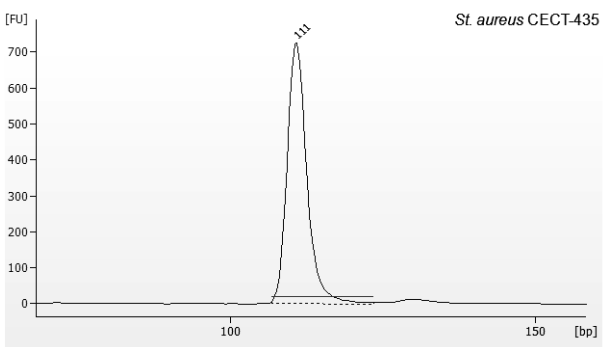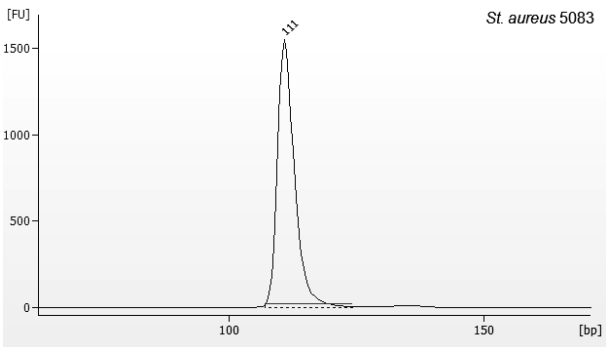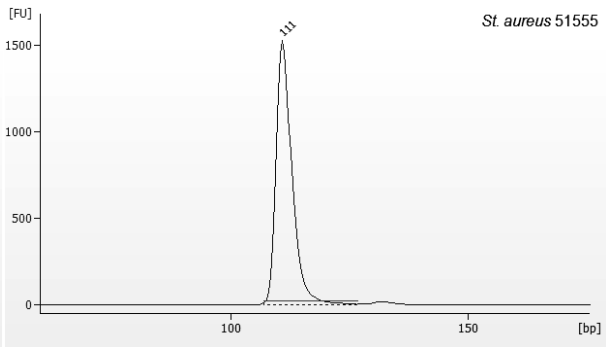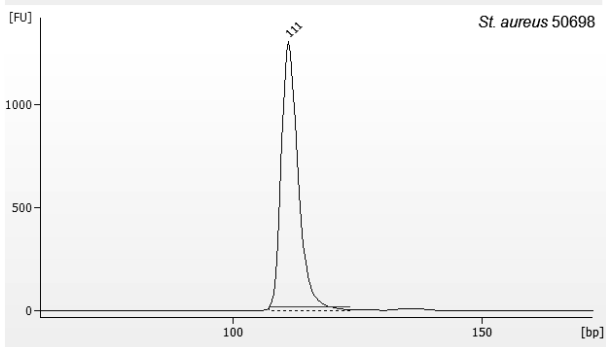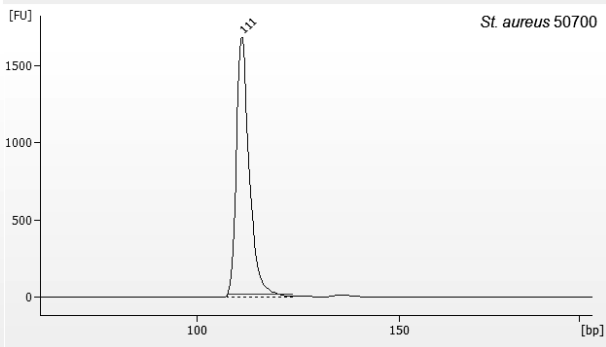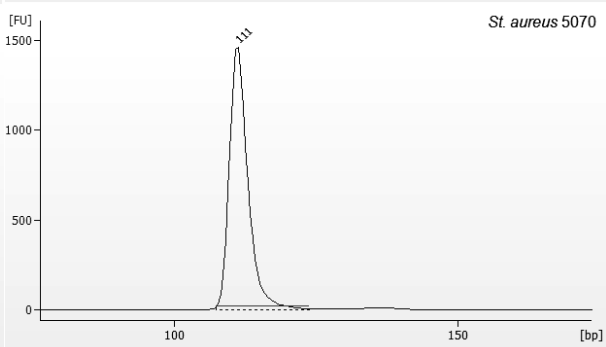

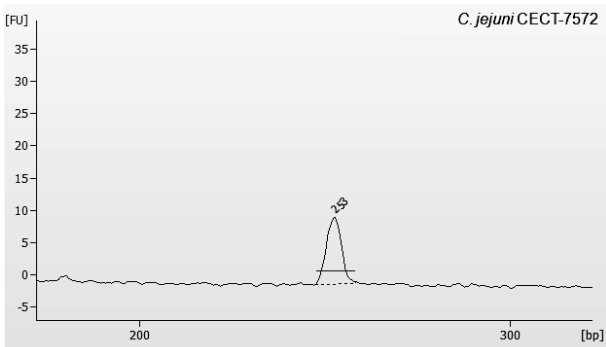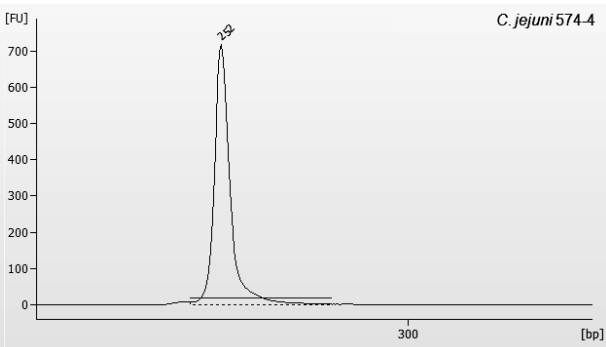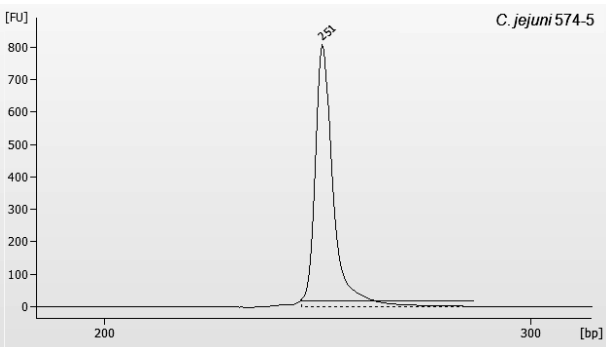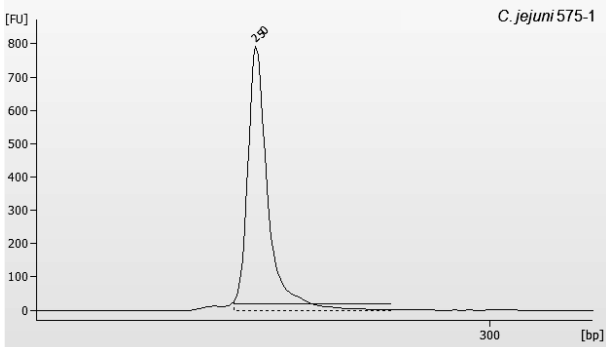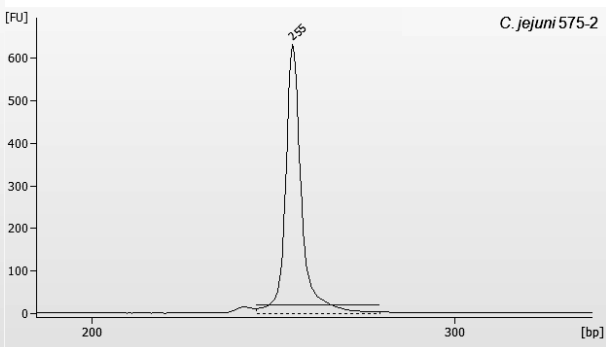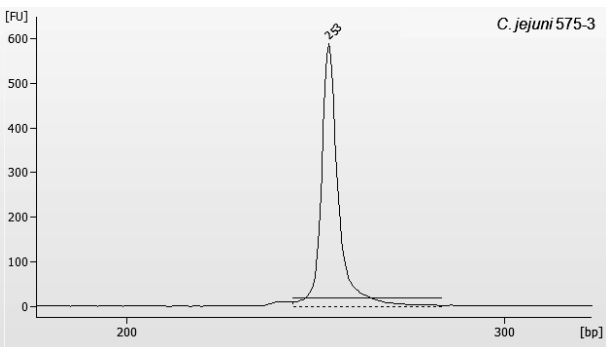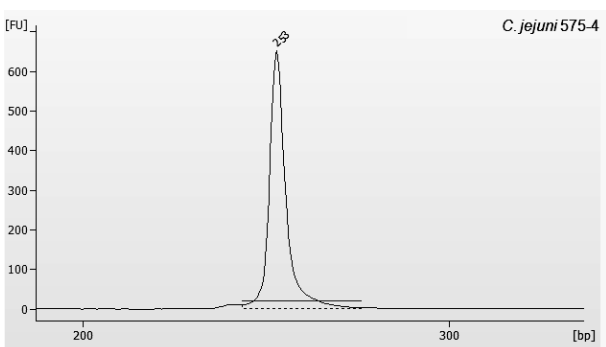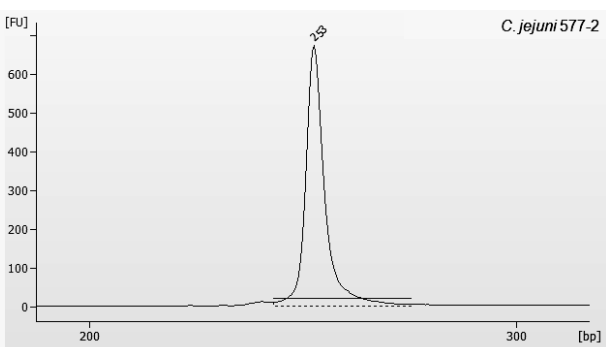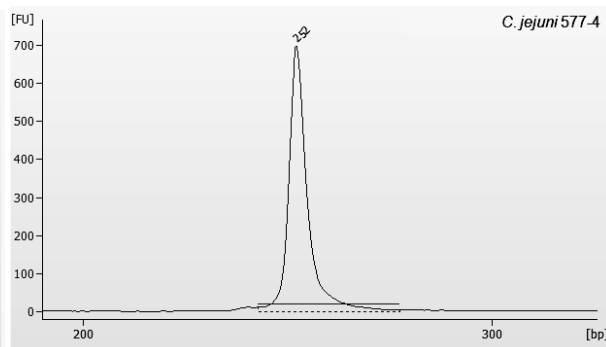

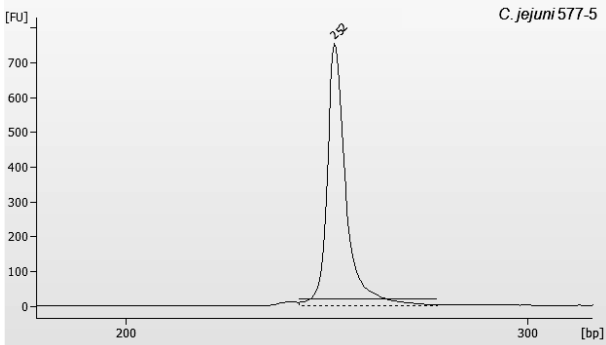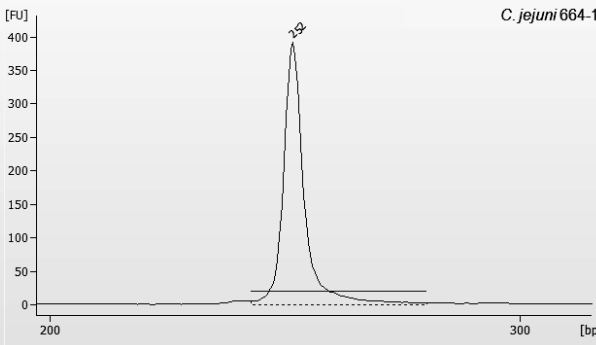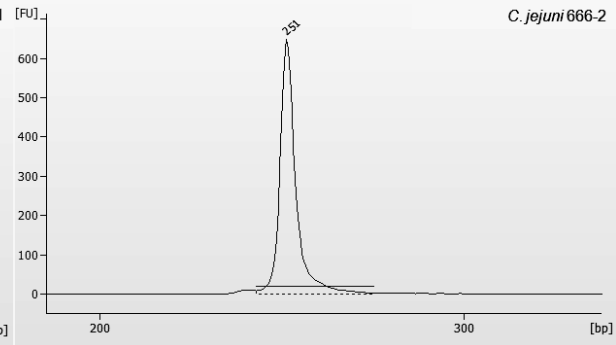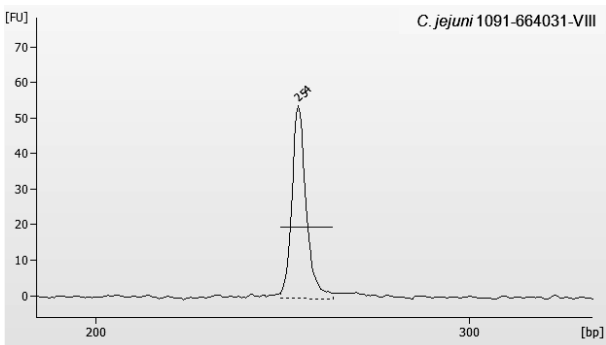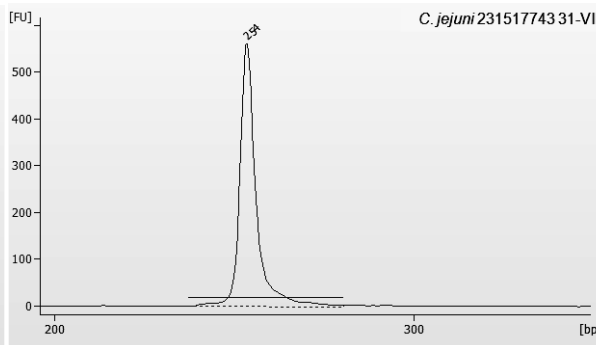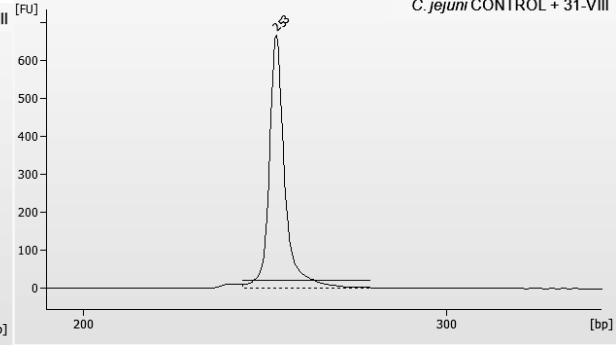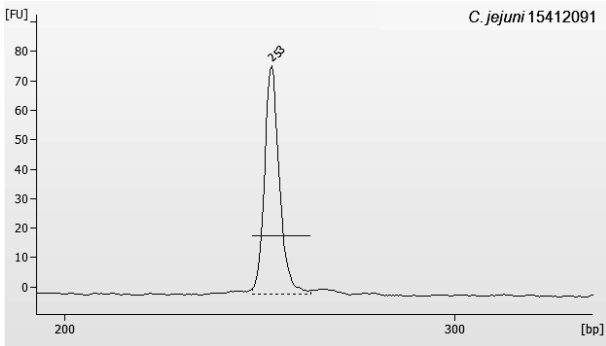

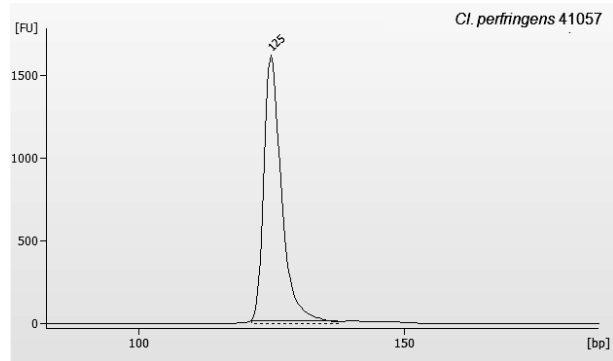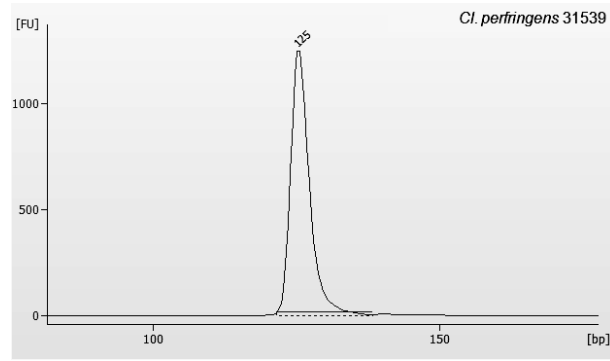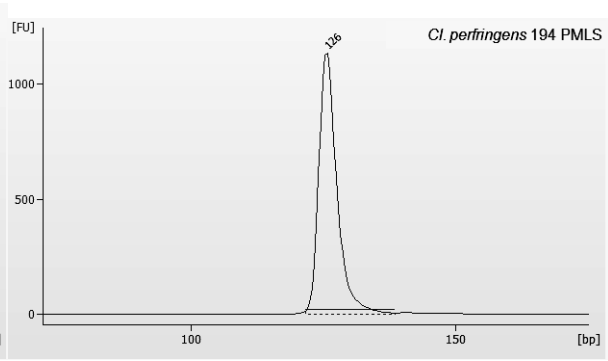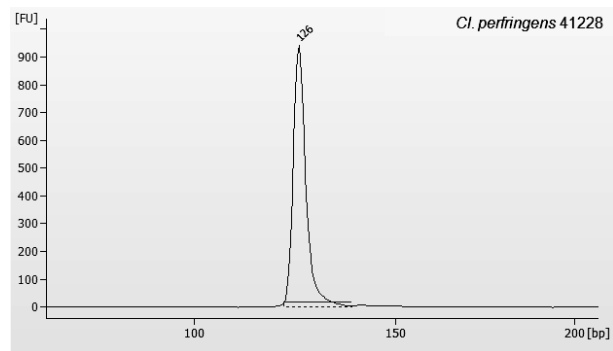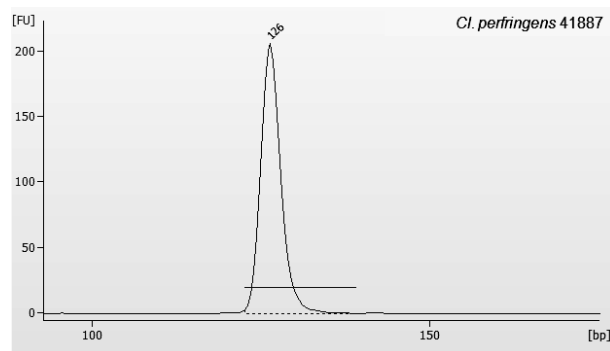

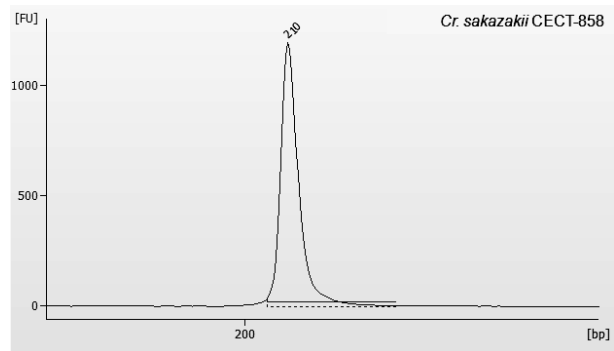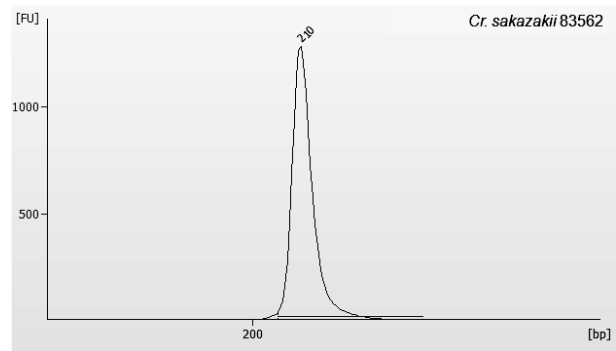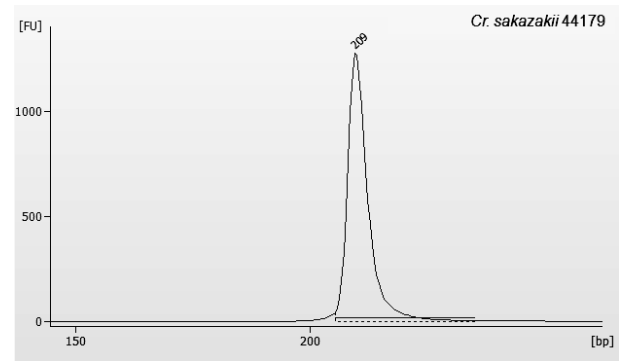

Supplement: Figure S2 — Amplicons obtained by mPCR for different strains of the tested bacterial species. Values showed on peak's top corresponds to the size detected for each amplicon in bp. B. cereus strains: CECT-193, 83535, 34588, 53591, 83536, 83537, HUCA-431. L. monocytogenes strains: CECT-7467, 41319, 31925, 883, 103545-4, 61318, HUCA-17. St. aureus strains: CECT-435, 5083, 51555, 50698, 50700, 5070. C. jejuni strains: CECT-7572, 574-4, 574-5, 575-1, 575-2, 575-3, 575-4, 577-2, 577-4, 577-5, 664-1, 666-2, 1091-664031-VIII, 23151774331-VIII, 31-VIII, 15412091. Cl. perfringens strains: 41057, 31539, 194PMLS, 41226, 41887. Cr. sakazakii strains: CECT-858, 83562, 44179. [file Presentation2.PDF]
